# A Fully ab Initio Kinetic Monte Carlo Approach for Modeling Adsorption and Diffusion in Interstellar Icy Grain Mantles: The Case of H_2_S

**DOI:** 10.1021/acsearthspacechem.5c00208

**Published:** 2025-12-18

**Authors:** Vittorio Bariosco, Stefano Pantaleone, Cecilia Ceccarelli, Piero Ugliengo, Albert Rimola

**Affiliations:** † Departament de Quimica, Universitat Autònoma de Barcelona, 08193 Bellaterra, Catalonia, Spain; ‡ Dipartimento di Chimica and Nanostructured Interfaces and Surfaces (NIS) Centre, 9314 Università degli Studi di Torino, via P. Giuria 7, 10125 Torino, Italy; § Institut de Planetologie et d’Astrophysique (IPAG) Université Grenoble Alpes, F-38000 Grenoble, France; ∥ Accademia delle Scienze di Torino, Via Maria Vittoria, 3, 10123 Torino, Italy

**Keywords:** astrochemistry, dense clouds, ISM matter, binding energies, diffusion barriers, kinetic Monte Carlo, diffusion coefficient

## Abstract

Understanding diffusion on interstellar ices is key to modeling the chemical evolution of cold molecular clouds, where low temperatures severely limit molecular mobility. In this study, we introduce a robust and fully automated multiscale computational framework to quantify diffusion processes of adsorbates at the surface of amorphous solid water (ASW). Using H_2_S as a test case, whose binding sites were previously studied at the ab initio level, we constructed a detailed network of 141 adsorption sites connected by over 270 transition states. All density functional energetics were benchmarked against DLPNO–CCSD­(T), achieving chemical accuracy in the description of diffusion barriers, which span from 0.1 to 27 kJ mol^–1^ with a median value of 5.4 kJ mol^–1^. An off-lattice kinetic Monte Carlo (kMC) model adopting both the ab initio diffusion barriers and binding energies for the desorption processes was carried out to compute temperature-dependent diffusion coefficients and to reconstruct the temperature-programmed desorption (TPD) curve. Our simulations reveal that thermal diffusion of H_2_S is negligible below 20 K, with diffusion coefficients as low as 10^–48^ cm^2^ s^–1^ at 10 K, thus excluding Langmuir–Hinshelwood surface encounters under typical dense cloud conditions. Moreover, under submonolayer conditions, diffusion was found to have negligible influence on the reconstructed TPD peak position. Furthermore, our results demonstrate that a universal scaling factor *f* to guess the diffusion barriers (Δ*E*_diff_) from the sole knowledge of BE: *f* = Δ*E*_diff_/BE does not apply as it exhibits wide variability across the sampled configurations. These findings highlight the need for incorporating statistically meaningful distributions of binding energies and diffusion barriers in astrochemical models to more accurately capture diffusion and surface reactivity on interstellar ices.

## Graphical abstract

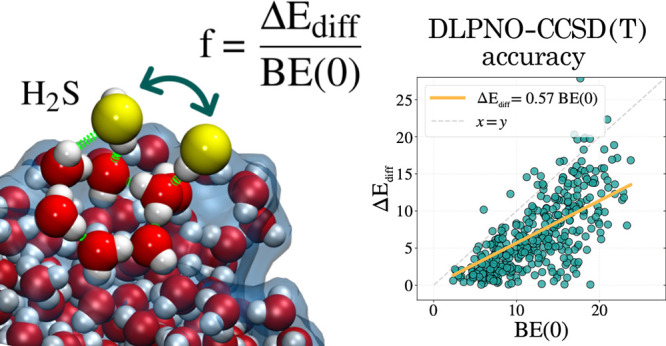

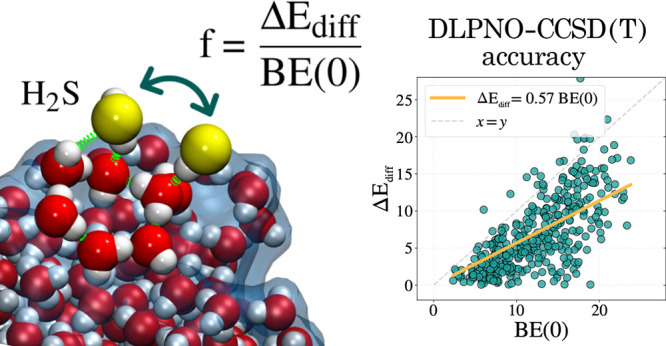

## 1 Introduction

The diffusion of atoms and molecules on interstellar dust grain surfaces plays a crucial role in the formation and evolution of interstellar ices and, possibly, complex organic molecules (iCOMs).^1–5^ For example, the thermally driven Langmuir–Hinshelwood mechanism is still predominantly advocated in the astrochemical models aiming to describe the formation of molecules on ices, including iCOMs,^6–10^ despite alternative mechanisms having recently been considered, such as the Eley–Rideal and ”hot-atom” ones^11–13^ or enhanced mobility in the CO-ice transition phase.^14,15^

In standard models that use the rate-equation formalism, the rate *R*_*AB*_ of the formation reaction from two reactant species *A* and *B* is expressed as *R*_*AB*_ = *k*_*AB*_ *n*_*A*_ *n*_*B*_, where *n*_*A*_ and *n*_*B*_ are the number density of the two species and *k*_*AB*_ is the rate constant at a given temperature. For surface reactions, the latter is given by:^6^

$$k_{AB}=\epsilon_{AB}\frac{R_{\mathrm{diff},A}+R_{\mathrm{diff},B}}{n_{d}}$$ 1

where ϵ_*AB*_ is an efficiency factor for the reaction to occur, *n*_*d*_ is the number density of the dust particles, and *R*_diff,*A*_ and *R*_diff,*B*_ are the diffusion rates for the species *A* and *B*, respectively. The diffusion rate *R*_diff_ of a species *x* is defined as 1/*t*_diff_, where *t*_diff_ is the time a species takes to scan the whole grain. The diffusion of species *A* and *B* also enters in the reaction efficiency, as follows:^16–18^

$$\epsilon_{AB}=\frac{k_{\mathrm{reac},AB}}{k_{\mathrm{reac},AB}+k_{\mathrm{diff},A}+k_{\mathrm{diff},B}+k_{\mathrm{des},A}+k_{\mathrm{des},B}}$$ 2

where *k*_reac,*AB*_ is the rate constant of the *A* + *B* reaction, *k*_diff,*A*_ and *k*_diff,*B*_ are the diffusion rate constants of species *A* and *B*, and *k*_des,*A*_ and *k*_des,*B*_ their desorption rate constants. Therefore, an accurate evaluation of the rates (desorption, reaction and diffusion) is fundamental to model the molecule formation on the interstellar dust grain surfaces.

In the past, a lot of attention has been devoted to estimate the reaction^18–23^ and desorption^24–31^ rates, while few studies focused on the diffusion.^32–38^ The major reason is the difficulty of measuring the diffusion rate in the laboratory, particularly so for atoms and radicals on amorphous solid water (ASW), where only a few specialized experimental setups have been developed to achieve this goal^32,34–36^ (see the review from Ligterink et al.^39^ for a comprehensive discussion). From a theoretical point of view, accurately determining diffusion barriers (as with any activation barrier) requires locating the corresponding transition state. This involves a computationally demanding, technically challenging, and difficult-to-automate procedure. Therefore, due to the lack of these data, barriers are usually assumed as a fixed fraction of the binding energy (BE), Δ*E*_diff_ = *f* × BE, with *f* varying from 0.3 to 0.8.^6,40–42^

To address the limited availability of reliable diffusion parameters for astrochemically relevant species, several laboratory studies have been conducted over the past decade.^32,34,35,43–49^

Kouchi et al.^46^ adopted a novel approach in the field using in situ ultrahigh-vacuum transmission electron microscope (UHV-TEM) to study the mobility of CO and CO_2_ on ASW, where the spatial distribution of crystalline islands was interpreted as a marker of diffusion. The same experimental setup was later adopted by Furuya et al.^48^ to study the diffusion of different molecules, including H_2_S.

Mispelaer et al.^34^ employed a bilayer approach, depositing a thin layer of the target molecule (H_2_CO, NH_3_, HNCO, CO) beneath ASW and tracking its diffusion through the ice matrix via infrared absorption decay at isothermal temperatures. This approach was subsequently employed and further developed by Lauck et al.^43^ and Maté et al.^47^ to investigate the diffusion of CO and CH_4_, respectively.

More recently, He et al.^49^ examined CO_2_ diffusion on ASW by tracking cluster formation via spectral changes during isothermal holds, fitting their temporal evolution with diffusion models to derive diffusion coefficients. Matar et al.^50^ deposited D atoms onto ASW and monitored the evolution of the adsorbed species by temperature-programmed desorption (TPD) coupled with quadrupole mass spectrometry, thus probing diffusion processes occurring at the ice surface. This approach has subsequently been applied to investigate the mobility of O and N atoms on ASW.^35,44^ However, the TPD technique is less suitable for characterizing irregular amorphous surfaces, where the activation energy cannot be represented by a single value but rather by a broad distribution.^39^ Finally, Watanabe et al.^32^ studied the diffusion of H on ASW adopting a combination of photostimulated desorption (PSD) with resonance-enhanced multiphoton ionization (REMPI). The same experimental setup was then adopted by Tsuge et al. to study C and hydroxyl radical diffusion on ASW.^36,51^ While these experimental efforts offer valuable insights into surface and bulk diffusion processes under astrophysical relevant conditions, they often suffer from intrinsic limitations, including the concurrent influence of desorption, structural changes in the ice during measurements, narrow temperature windows. Additionally, many studies determine only the diffusion barrier while assuming a fixed prefactor, rather than measuring both simultaneously.^45,49^

On the computational side, comparatively fewer studies have addressed diffusion processes on interstellar ices. The basic challenge lies in the rare-event nature of diffusion at the extremely low temperatures characteristic of environments where interstellar ices are formed (∼10 to 20 K). In such conditions, thermally activated hopping exhibit Δ*E*_diff_ ≈ 400–500 K (3–4 kJ mol^–1^), meaning they occur over time scales of years, far exceeding the accessible window of classical or ab initio molecular dynamics (AIMD). Consequently, simulating these rare events using conventional AIMD based on Density Functional Theory (DFT) becomes computationally prohibitive, owing to the exceedingly long time scales required for diffusion to occur. While enhanced sampling techniques such as metadynamics can mitigate this limitation, they rely on the careful and often system-specific definition of collective variables for each of the diffusive path of the entire manifold. To circumvent these limitations and extend the accessible time scales, kinetic Monte Carlo (kMC) methods are often employed. These simulations require precomputed BE and Δ*E*_diff_ for all relevant adsorption sites and transition pathways, which serve as input to the kMC engine. These approaches allow for the stochastic sampling of rare diffusion events and the modeling of long-time scale structural evolution, effectively bridging the gap between atomistic dynamics and macroscopic behavior.^42,52^

Karssemeijer et al. carried out a series of studies on the diffusion of CO and CO_2_ on ASW surfaces.^53–55^ To explore the potential energy surface (PES), they employed an off-lattice adaptive kinetic Monte Carlo (AkMC) approach, where all possible diffusion events and their transition states are determined on-the-fly during the simulation using classical pair potentials for H_2_O–CO interactions. This allows identifying all relevant diffusion pathways and their associated transition states. Once the relevant diffusion rates were identified, an on-lattice kMC simulation was performed to model the long-time scale evolution of molecular diffusion on the ASW surface.

Ásgeirsson et al.^56^ computed H atom adsorption and diffusion on both crystalline and amorphous ice surfaces using the analytic H–H_2_O interaction potential developed by Andersson et al.,^57^ which was parametrized from ab initio data for the H_2_O + H ⇄ OH + H_2_ reaction. Diffusion barriers were identified using nudged elastic band (NEB) and minimum-mode following methods, and the derived rate constants were used in kMC simulations to compute temperature-dependent diffusion coefficients over long time scales. Senevirathne et al.^58^ subsequently adopted the same H–H_2_O potential to study H atom diffusion on larger crystalline and amorphous ice models, where the water molecules were kept fixed. By adopting the NEB method, they systematically mapped all local minima and transition states on the PES. More recently, Zaverkin et al.^59^ investigated the diffusion of atomic nitrogen on ASW using a neural network potential trained on reference structures and energies computed at the PBEh-3c/def2-mSVP level. The enhanced sampling via metadynamics allowed for a thorough exploration of the PES, resulting in the identification of a statistically meaningful number of minima and transition states. The derived landscape was subsequently incorporated into an on-lattice kMC framework to simulate long-time scale diffusion dynamics at DFT-level accuracy.

While these approaches have provided valuable insights for the astrochemical community, they remain fundamentally limited by the accuracy of the underlying PES, which are either based on force fields or, at best, trained to reproduce DFT-level energetics. This upper bound in accuracy poses a critical limitation: in weakly bound systems, diffusion barriers often lie close to, or below, the threshold of chemical accuracy (∼5 kJ mol ^–1^),^55,56^ making them particularly sensitive to small errors in the PES. As a result, to reliably capture such subtle energetic differences, highly accurate methods that go beyond standard DFT are required, such as the CCSD­(T), widely regarded as the gold standard for chemical accuracy.^60^

In this work, we relied on the results from our previous paper on the BE distribution of H_2_S adsorbed on a large (200 H_2_O molecules) amorphous icy grain model,^29^ created by the ACO-FROST^61^ program. The 141 minima obtained through our ACO-FROST ice grain were connected to build a diffusive network, ending up with almost 300 transition state structures. All the energies from high-quality DFT are benchmarked and corrected at DLPNO–CCSD­(T) level, ensuring reliable values close to the chemical accuracy. Subsequently, the set of binding sites and diffusion barriers was fed into an ad hoc kMC model to calculate diffusion coefficients at different increasing temperatures taking into account the possibility of desorption events. Finally, we investigated the effect of diffusion on the simulated Temperature-Programmed Desorption (TPD) spectrum, the primary observable in experimental desorption studies, an aspect that was not accounted for in our previous work.^29^

The article is organized as follows. Section 2 describes the procedure used to construct the diffusive network and outlines the computational methodology. Section 3 presents the results and discussion, covering both the ab initio calculations and the kMC simulations. Finally, Section 4 summarizes the major findings and concludes the article.

## 2 Methodology

### 2.1 Computational Methods

All the calculations reported in this work are carried out using the ORCA program (v. 5.0.3)^62^ on the cluster model representing an amorphous ice grain mantle described in the next section. The ONIOM (Our own N-layered Integrated molecular Orbital) approach is used for optimizations, frequency calculations (carried out within the harmonic approximation to calculate the zero-point energy (ZPE) correction) and minimum energy path (MEP) optimization.^63^ Within the ONIOM method, the system is divided into two fictitious subsystems: (i) the Real Zone, which represents the entire system and is treated using a lower level of theory (e.g., semiempirical quantum mechanics (SQM) or a less accurate quantum mechanical method (QM2)); and (ii) the Model Zone, which is a smaller, crucial part of the system, including key interactions (such as the adsorbate and surrounding water molecules), and is treated with a higher quantum mechanical (QM) level of theory. The final ONIOM (QM:SQM) energy for each structure is calculated as

$$E_{\mathrm{ONIOM}}=E_{\mathrm{Real}}^{\mathrm{SQM}}-E_{\mathrm{Model}}^{\mathrm{SQM}}+E_{\mathrm{Model}}^{\mathrm{QM}}$$ 3

The *x*TB-GFN2 method is used in all the ONIOM calculations as the SQM low level for its accuracy at reasonable cost to describe water–water interaction.^64,65^ The B97–3c functional is used as QM method for the high-level calculation.^66^ This functional has been recently benchmarked on the interaction between H_2_O and H_2_S.^29^ The energies are then refined applying a correction factor obtained at DLPNO–CCSD­(T) level (see SI Section 2 for details). The atoms outside the Model region are kept fixed, with the Model zone embedded electrostatically using the default settings. Tight convergence criteria were applied to both the SCF and geometry optimization, while the integral evaluation grid was set to the highest density (using the keywords !tightSCF, !tightOPT, and !defgrid3 in the ORCA program). In cases where imaginary frequencies were detected, the geometry optimization was restarted with an even tighter convergence criterion (!verytightOPT), which generally led to the correct minima.

The identified diffusive paths were scanned using the climbing image nudged elastic band (CI-NEB) method, with 5 or 8 images chosen based on the distance between the two binding sites. The initial images (i.e., reactant and product) were interpolated using the image-dependent pair potential (IDPP) algorithm.^67^ This method prevents atomic overlap and generates a path that is closer to the MEP with respect to the linear interpolation in Cartesian coordinates. The convergence of the Band is monitored through the Climbed Image (CI) only. To optimize the MEP, the FIRE algorithm^68^ is employed. Finally, once the highest energy is identified, a frequency calculation is performed to verify if it corresponds to a true Transition State (TS), indicated by the presence of one eigenvector with a negative eigenvalue. Otherwise, the TS is optimized (in 50 out of 273 cases) giving as input the previous frequency calculation and setting the convergence to tight (!OptTs, Convergence tight in Section %geom).

#### 2.1.1 Diffusion Rate and Desorption Rate

Diffusion barrier (Δ*E*_diff_) represents the energy difference between the TS and the local minima along the reaction path. As we are dealing with diffusion, we do not arbitrary define reactants and products, but only two generic minima. This allowed us to increase the statistics of diffusive barriers, by considering both the forward (min1) and backward (min2) ”reactions”:

$$\Delta E_{\mathrm{diff},\mathrm{min1}}=E_{\mathrm{TS}}^{0}-E_{\min1}^{0}\;\mathrm{and}\;\Delta E_{\mathrm{diff},\mathrm{min2}}=E_{\mathrm{TS}}^{0}-E_{\min2}^{0}$$ 4

where *E*_min1_^0^, *E*_min2_^0^, and *E*_TS_^0^ are the ONIOM ZPE-corrected energies for the two minima and the transition state, respectively. To make the notation less cumbersome, in the following we will generically refer to each diffusion barrier as Δ*E*_diff_, irrespective of the backward/foreward direction.

The final diffusion constant *k*_diff_ is computed using the Eyring equation:

$$k_{\mathrm{diff}}(T)=\nu_{\mathrm{diff}}\exp\left(-\frac{\Delta E_{\mathrm{diff}}}{RT}\right)$$ 5

where ν_diff_ is the diffusion prefactor, computed according to the transition state theory:

$$\nu_{\mathrm{diff}}∼\nu_{\mathrm{TST}}(T)=\frac{k_{B}T}{h}\frac{q_{\mathrm{vib}}^{‡}}{q_{\mathrm{vib}}}$$ 6

here, *k*_B_ is the Boltzmann constant, *h* the Planck constant and *q*_vib_^‡^ and *q*_vib_ are the partition functions of transition state and min1 (reactant). The prefactor represents the attempt frequency to reach the product from the reactant, here approximated with the imaginary frequency (ν_diff_) defined from the negative eigenvalue associated with the translational mode driving the reactant toward the product. This approximation introduces deviations in the mean value of up to 1 order of magnitude at 10 K (ν_TST_ = 2.1 × 10^11^ s^–1^, ν_diff_ = 3.2 × 10^12^ s^–1^), decreasing to less than a factor of 2 at 50 K (ν_TST_ = 1.3 × 10^12^ s^–1^).

Equation 4 along with eqs 5 and 6, ensure microscopic reversibility as done in Cuppen and Hornekaer^69^ (see Figure S5 in the Supporting Information).

The desorption energy is defined as a common BE value corrected for the ZPE energy:

$$\mathrm{BE}(0)=-\Delta E=E_{\mathrm{ads}}^{\mathrm{iso}}+E_{\mathrm{gra}}^{\mathrm{iso}}-E_{c}-\Delta \mathrm{ZPE}$$ 7

where (*E*_c_), (*E*_ads_^iso^) and (*E*_gra_^iso^) are the total energies of the complex, and the isolated adsorbate and grain, respectively. The ΔZPE is defined as

$$\Delta \mathrm{ZPE}={\mathrm{ZPE}}_{c}-{\mathrm{ZPE}}_{\mathrm{ads}}^{\mathrm{iso}}-{\mathrm{ZPE}}_{\mathrm{gra}}^{\mathrm{iso}}$$ 8

In a similar fashion to the *k*_diff_, the desorption kinetic constant (*k*_des_) is computed with the Arrhenius expression:

$$k_{\mathrm{des}}\left(T\right)=\nu_{\mathrm{des}}\exp\left(-\frac{\mathrm{BE(0})}{\mathrm{RT}}\right)$$ 9

where the desorption prefactor ν_des_ is computed using the formula from Tait et al.,^70^ already used in our previous work.^29^

### 2.2 Strategy to Identify Effective Diffusive Path

The icy grain model and the binding site geometries were taken from the previous work by some of us.^29^ Out of the 141 binding sites, we only connect to each other those within a maximum cutoff threshold distance of 5.1 Å, computed from the positions of the sulfur atoms. This procedure brings 410 unique initial diffusive paths. The workflow to identify the effective diffusive path is as follows:

1. To avoid double counting, we considered the unidirectional diffusion, i.e. if binding site 1 goes into binding site 2, then 2 cannot go into 1. Then, for each jump one TS is identified with the two associated diffusion barriers (Δ*E*_diff,min1_ and Δ*E*_diff,min2_), see Section 2.1.1);
2. All the diffusive path lengths below 1 Å are discarded (36 cases identified);
3. Among the 374 remaining cases, those where H_2_S acts as both H-bond acceptor and donor with the same water molecule were excluded. This condition, together with the previous one, avoids considering rotational barriers (21 cases identified). Figure 1 shows a case study of a diffusive path connected by a rotational motion;
4. The ONIOM QM:SQM method^63^ is applied to reoptimize the reactant and product geometries. This is due to the change of the Model zone; indeed, the new Model zone includes all atoms within the sum of the two 5 Å regions around each connected binding sites. A sketched example is shown in Figure 2A to illustrate how the new model zone is created. Finally, the same method is applied also to optimize the MEP.
5. A topological continuity criterion is set in order to avoid jumps between sites without any overlap of the respective model zones. As depicted in Figure 2B, if the two Model zones do not overlap, the diffusive path is discarded (10 cases found);
6. Due to the increased model zone, the starting and end points, hereafter referred to as reactant and product respectively, are reoptimized with the enlarged zone along with a frequency calculation to check if they are real minima (see SI Section 3 for details);
7. Step 3 is repeated to check if the geometry changed after the reoptimization (15 cases discarded);
8. On the final 328 diffusive paths, TS geometries were searched using the CI-NEB technique implemented in ORCA.^62,71^ From this set, 55 structures were excluded due to presence of intermediate minima, giving back 273 paths;

**Figure 1:**
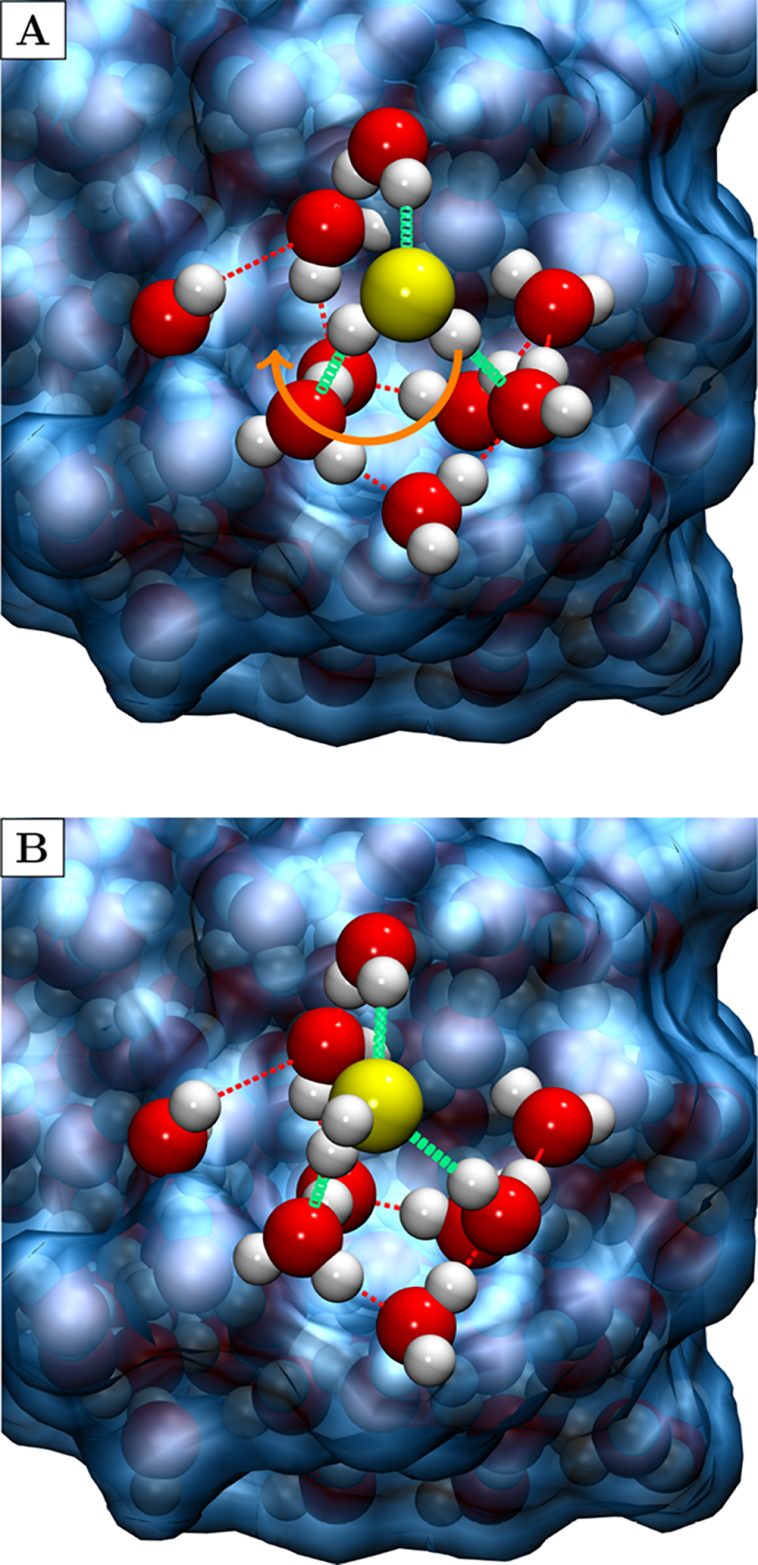
Example of two minima connected by a rotational barrier, where H_2_S forms H-bonds with the same water molecules: (A) two as the donor and one as the acceptor; (B) one as the donor and two as the acceptor. The orange arrow indicates the rotation connecting the two minima. The icy-blue background illustrates the grain surface not included in the MEP optimization. Color-coding: white, H atoms; red, O atoms; and yellow, S atom. Pictures made with VMD^72^ and rendered using the Tachyon library.^73^

**Figure 2:**
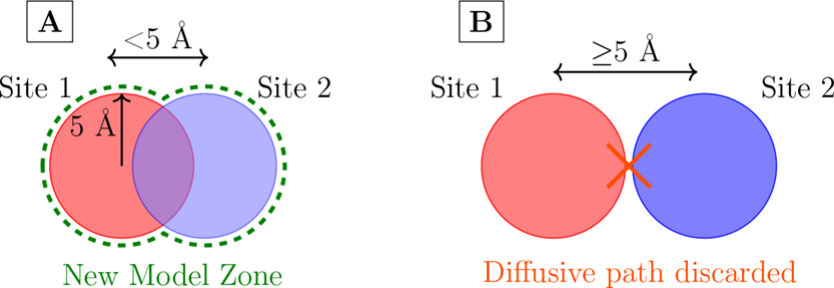
Simplified representation of the geometrical selection process for diffusive paths. (A) Two model zones from distinct binding sites (i.e., Site 1 and Site 2) share some water molecules in the zone of overlap. The new model zone (indicated by the dashed green line) represents the combined areas of both model zones. (B) When the model zones do not overlap, the diffusive path is discarded.

### 2.3 Diffusive Network

A static analysis of inherently dynamic phenomena, such as diffusion, requires careful benchmarking to mitigate potential biases in the methodology. While a dynamic approach allows the system to explore various conformations and positions on the surface, the static method relies on the initial sampling of minima and transition states, which serves as the foundation for kMC simulations.^74^ A key factor in the static analysis of diffusion in a system is the capability of individual molecules to move freely across the grid. To assess the mobility of each molecule on the grid, we incorporate concepts from graph theory.^75^ In the Supporting Information a detailed description of the procedure applied to obtain a physically meaningful diffusion network is reported.

### 2.4 Kinetic Monte Carlo Model

We developed the kMC code considering explicitly diffusion barriers and desorption energies computed in this work. We adopted the BKL algorithm, or ”n-fold way”, as presented in the original paper by Bortz, Kalos and Lebowitz.^76,77^ This procedure is also defined as ”rejection-free”, meaning that even at very low temperature and with high energy barrier, the system will undergo a diffusion or desorption event. When desorption occurs, the kMC run stops and resumes from the initial BE site until the maximum number of iterations for that BE site is reached. The adopted code is fully available online (see Section 4).

We ran 10 different simulations for each BE sites found on the surface, for a total of 1310 independent simulations. We repeated this for all the explored range of temperatures, defining a finite ramp of temperature from 10 K up to 80 K, increasing every 5 K. This gives back 15 kMC runs at different temperatures. The total number of runs is therefore 19650.

For each run, two independent cutoff criteria were used, and the simulation was stopped when either one was met. The first criterion is based on the number of iterations, with an upper limit of 10^9^ steps.^56,59^ The second criterion was based on the total diffusion time, set to 600 s (10 min), corresponding to a typical experimental time scale and preventing the system from evolving over durations not relevant to laboratory conditions. Time is updated following the n-fold way as

$$\Delta t=-\frac{\ln\left(r\right)}{R}$$ 10

where Δ*t* is the stochastic time increment associated with the occurrence of the selected event, *r* is a random number drawn from a uniform distribution in the interval (0, 1] and *R* = ∑_*i*_ *r*_*i*_ is the total rate, given by the sum of the rates *r*_*i*_ of all possible events *i* in the specific binding site.

The diffusion coefficient was calculated from the mean squared displacement (MSD) of H_2_S, using its center mass and by time averaging along each discrete jump:

$$D\left(T\right)=\underset{i}{\sum }\frac{D_{i}\left(T\right)\Delta t_{i}}{t}$$ 11

where *D*(*T*) is the diffusion coefficient obtained from an independent kMC trajectory, Δ*t*_*i*_ is the residence time of the *i*-th diffusion event, and *t* is the total simulation time. In this formulation, the contribution of each event is weighted by its duration, allowing a proper time average of the diffusion coefficient rather than relying on an instantaneous value. The diffusion coefficient for a single event, *D*_*i*_(*T*), is defined as

$$D_{i}\left(T\right)=\frac{{\left(r\left(t_{i}\right)-r\left(t_{i-1}\right)\right)}^{2}}{2d\Delta t_{i}}$$ 12

where *r* represents the center of mass of the H_2_S molecule, approximated here by the position of the sulfur atom. The parameters *t*_*i*_ and *t*_*i*–1_ refer to the current and previous KMC steps, respectively, and *d* is the dimensionality of the system, set to 3.

Alternatively, one can estimate the diffusion coefficient by adopting the classical Arrhenius expression as

$$D^{\mathrm{median}}=D_{0}^{\mathrm{median}}\exp\left(-\frac{\Delta E_{\mathrm{diff}}^{\mathrm{median}}}{\mathrm{RT}}\right)$$ 13

the diffusion prefactor (*D*_0_) can be approximated adopting the equation:^78–81^

$$D_{0}^{\mathrm{median}}=\frac{na_{0}^{2}}{2d}\nu$$ 14

where *n* is the number of directly connected nodes, *a*_0_ is the average jump distance, *d* is the dimensionality of the system and ν is the prefactor whose value will be described in the next section. For our system *n* is equal to 3 and *d* is set to 3.

## 3 Results and Discussion

### 3.1 Ab Initio Results

Following the application of the selection criteria outlined in Section 2.2 and the exclusion of nodes disconnected from the network (see SI Section 1), the final number of nodes and edges is 133 and 370 respectively. Figure 3 presents the complete grid, with nodes and edges colored according to their BE(0) and Δ*E*_diff_ values, respectively.

**Figure 3:**
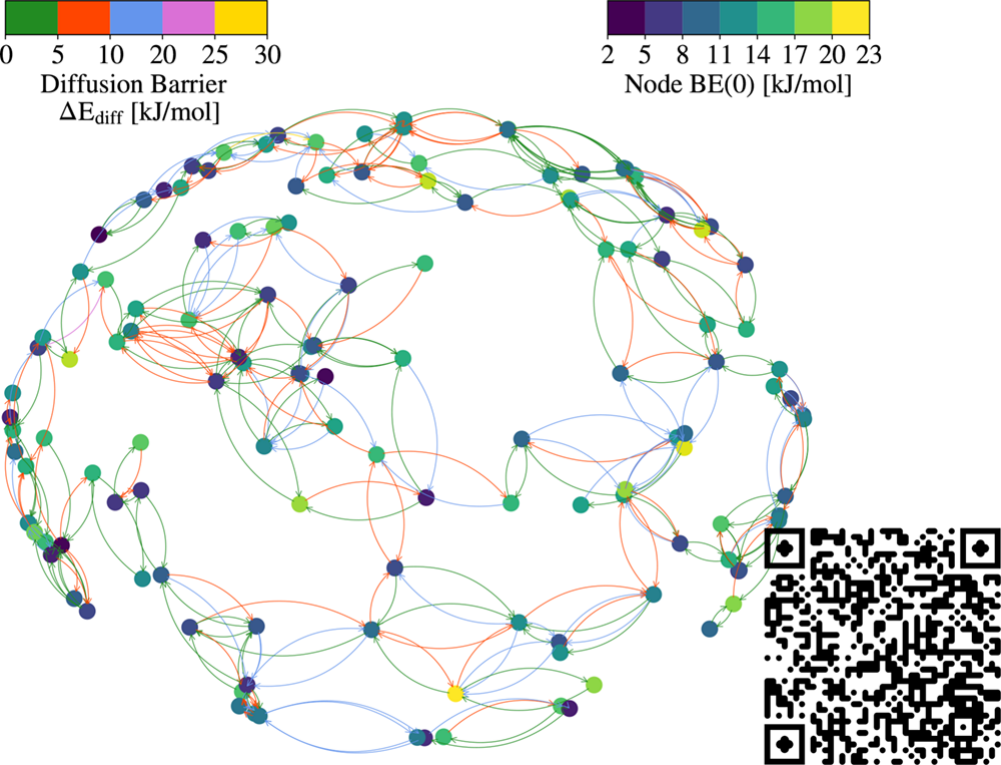
Final grid used for the kMC simulations, consisting of 133 nodes and 370 edges. The colormap on the left represents the Δ*E*_diff_ values calculated from Equation S7, which are used to color the edges connecting the nodes. In contrast, the colormap on the right shows the BE(0) values computed via Equation S8 and is used to color the nodes. The 3D version can be visualized by scanning the QR code or by clicking this link https://viba97.github.io/H2S_diffusion_3D_visualization/.

Figure 4A illustrates the distribution of diffusion barriers computed using Equation S7. The values span a broad range, from 0.1 to 27 kJ mol^–1^, exhibiting a pronounced right-skewed distribution. Given this asymmetric nature, the median value of 5.4 kJ mol^–1^ provides a better representation of the central tendency, as it is less sensitive to the extreme values in the tail of the distribution.^82^ The interquartile range (IQR) of 7.0 kJ mol^–1^, defined as the difference between the third quartile (Q3 = 9.7 kJmol^–1^) and first quartile (Q1 = 2.6 kJ mol^–1^), captures the spread of the central 50% of the data. Notably, a substantial fraction of diffusion events (25% below Q1) occur with barriers below 2.6 kJ mol^–1^, suggesting that a significant portion of the underlying PES is relatively flat with minimal energetic hindrance to diffusion. This finding is consistent with previous computational studies on weakly bound species such as CO, H, and N, which report diffusion barriers around or even below the threshold of chemical accuracy.^53,56,59^ Such a landscape also underscores the importance of employing accurate and well-calibrated computational methods, capable of resolving energy differences in the sub-kJ mol^–1^ range to reliably capture the dynamics of surface diffusion (see SI Section 3 for details).

**Figure 4:**
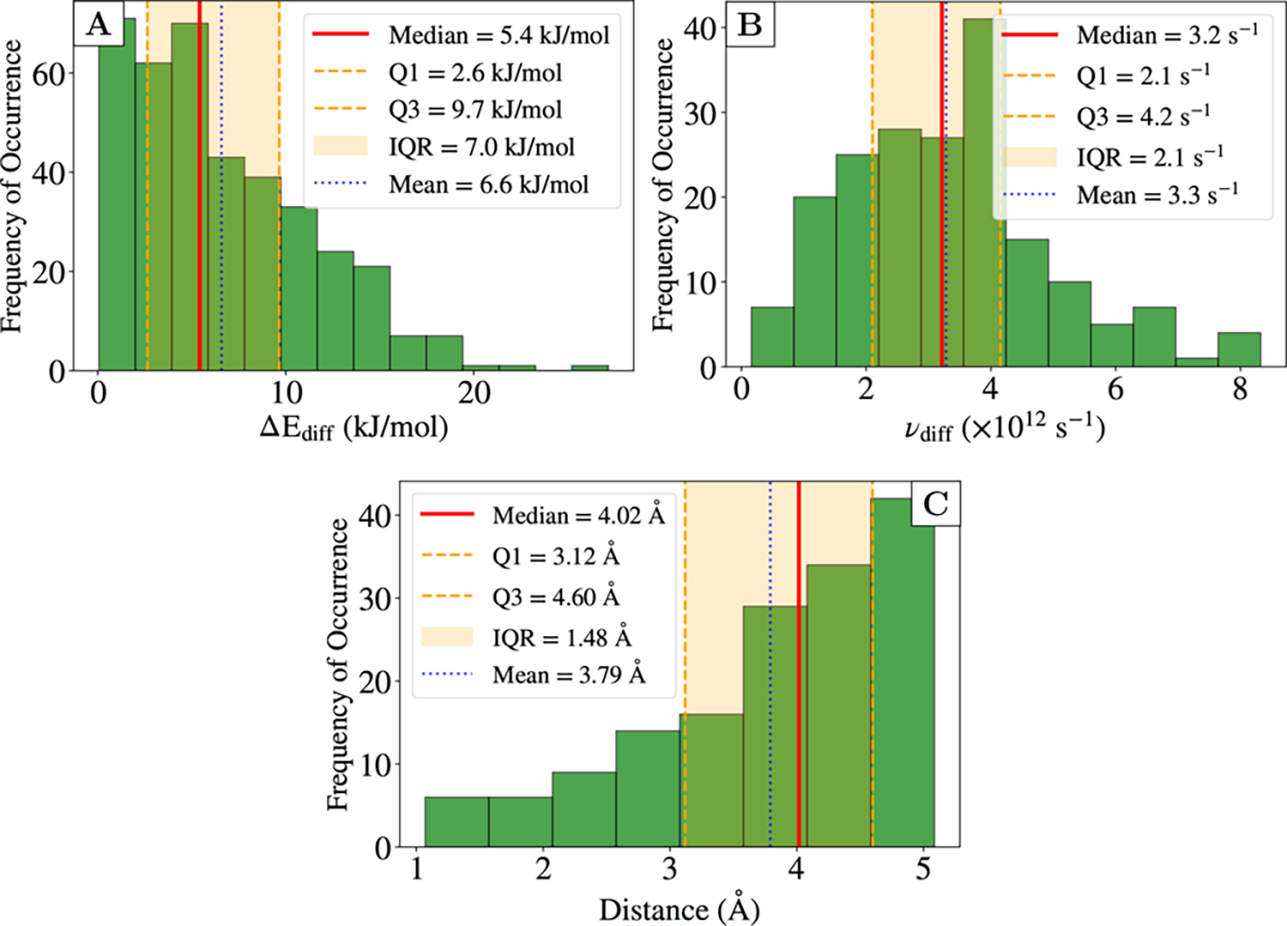
Histogram plots of (A) diffusion barrier distribution (in kJ mol^–1^), (B) ν_diff_ (in *s* ^–1^), and (C) distance distribution among connected nodes for the grid reported in Figure 3. The shaded orange band represents the interquartile range IQR, which contains the central 50% of the data. The number of bins and their width are obtained following the Freedman Diaconis estimator.^83^ The legend for the plot B is shortened for ease of visualization.

In Table 1, we report the diffusion prefactors (ν_diff_), diffusion coefficient prefactors (*D*_0_), binding energies (BE(0)) and diffusion barriers (Δ*E*_diff_) from this work, along with CO_2_, CO, and CH_4_ drawn from the literature. The inclusion of CO_2_ is motivated by its comparable binding energy range on ASW to that of H_2_S, as shown by Ferrero et al. (CO_2_: 12.4–24.5 kJ mol^–1^; H_2_S: 19.1–27.8 kJ mol^–1^),^24^ and by the similar desorption temperatures observed for both species in TPD experiments.^84^ Although CO and CH_4_ differ more significantly in binding and diffusion energetics, and, accordingly, direct one-to-one comparison should be made with caution, they are included to provide a broader context and to facilitate a qualitative comparison across astrochemically relevant species. Table 1 includes the only study to date specifically addressing the H_2_S diffusion, conducted experimentally by Furuya et al.,^48^ who reported an activation energy Δ*E*_diff_ value of 7.2 kJ mol^–1^. This value is in agreement with the median diffusion barrier obtained in our calculations (5.4 ± 3.5 kJ mol^–1^, see Figure 4A). However, it is worth noting that the approach adopted by Furuya et al. treats the diffusion activation energy of each species as a single fixed value per simulation, rather than considering a distribution of barriers arising from surface heterogeneity. As a result, potential contributions from low-barrier pathways, which may play a significant role in enhancing surface mobility, could be underestimated.

**Table 1:** Diffusion Prefactors (ν_diff_ in s^–1^), Diffusion Coefficient Prefactors (*D*_0_ in cm^2^ s^–1^), and Binding and Diffusion Barriers (BE(0) and Δ*E*_diff_ in kJ mol^–1^; Corresponding *K* Values in Parentheses) for H_2_S (from This Work and the Literature) and CO, CH_4_, and CO_2_ (from the Literature)^a^. Comp-DFT are data obtained from averaged DFT values, while Comp-kMC when including kMC simulations

species	ν_diff_	*D*_0_	BE(0)	Δ*E*_diff_	result	ref.^b^
CO	1.5 × 10^9^	3.0 × 10^–7^	7.2–13.3	4.1	Exp	^1^
			(870–1600)	(490)		
CO		5.0 × 10^–2^	14.9	4.8	Comp	^2^
			(1787)	(580)		
CH_4_	1.0 × 10^9^	1.0 × 10^–8^	8.2	4.0–5.3	Exp	^3^
			(990)	(477–639)		
CH_4_	2.6 × 10^9^	1.0 × 10^–7^	9.1–13.3	4.5	Exp	^1^
			(1100–1600)	(547)		
CO_2_		1.8 × 10^–2^	28.9	12.3	Comp	^4^
			(3470)	(1474)		
CO_2_	1.0 × 10^12^	1.0 × 10^–4^	18.9^c^	12.5	Exp	^5^
			(2267)	(1500)		
CO_2_	1.0 × 10^7.6^	1.0 × 10^–8^	18.7^d^	10.8	Exp	^6^
			(2250)	(1300)		
H_2_S	1.0 × 10^12^		19.1 ± 0.7^e^	7.2 ± 1.1	Exp	^7^
			(2296 ± 90)	(870 ± 130)		
H_2_S	3.2 ± 1.1 × 10^12^	3.9 ± 2 × 10^–3^	12.4 ± 4.9	5.4 ± 3.5	Comp-DFT	^8^
			(1497 ± 589)	(652 ± 421)		
H_2_S		6.3 ± 2.0 × 10^–3^		8.6 ± 0.2	Comp-kMC	^8^
				(1034 ± 24)		

a Comp-DFT are data obtained from averaged DFT values, while Comp-kMC when including kMC simulations.

b References: [1] He et al.;^45^ [2] Karssemeijer & Cuppen;^53^ [3] Mate et al.;^47^ [4] Karssemeijer & Cuppen;^55^ [5] Kouchi et al.;^46^ [6] He et al.;^49^ [7] Furuya et al.;^48^ [8] this work

c Noble et al.^85^

d He et al.^86^

e Penteado et al.^87^

Furthermore, in Table 1 and Figure 4B, we report the median value of the distribution of ν_diff_ adopted in this work. Physically, it reflects the characteristic attempt hopping frequency of the adsorbate from one minimum to another. In this work, we estimated the ν_diff_ using the imaginary frequency associated with the reaction coordinate at the transition state. This value typically falls in the range of 10^12^ s^–1^, according to the common expression as reported by Hasegawa and Herbst,^6^ and widely adopted in the astrochemical community. Nevertheless, as highlighted by He et al.,^49^ it is common practice to assume a fixed value of ν_diff_ while experimentally determining only Δ*E*_diff_. This simplification, though convenient, can significantly influence the extracted value of the diffusion barrier. A clear example of this is found in the comparison between the results of He et al.^86^ and He et al.:^49^ the former assumed a fixed ν_diff_ = 10^12^ s^–1^, leading to an estimated Δ*E*_diff_ = 2100 K, whereas the latter performed a simultaneous fit of both parameters, obtaining ν_diff_ = 10^7.6^ s^–1^ and *E*_diff_ = 1300 K. This discrepancy suggests that the *E*_diff_ reported by Furuya et al.,^48^ which was derived under the assumption of a fixed prefactor, may also be overestimated.

Figure 5 shows the correlation between our calculated BE(0) and the diffusion barrier Δ*E*_diff_. In astrochemical models, the *f*-Ratio = $\frac{\Delta E_{\mathrm{diff}}}{\mathrm{BE}}$ is typically assumed to lie between 0.3 and 0.8 for H_2_S.^6,8,40,48,88–91^

**Figure 5:**
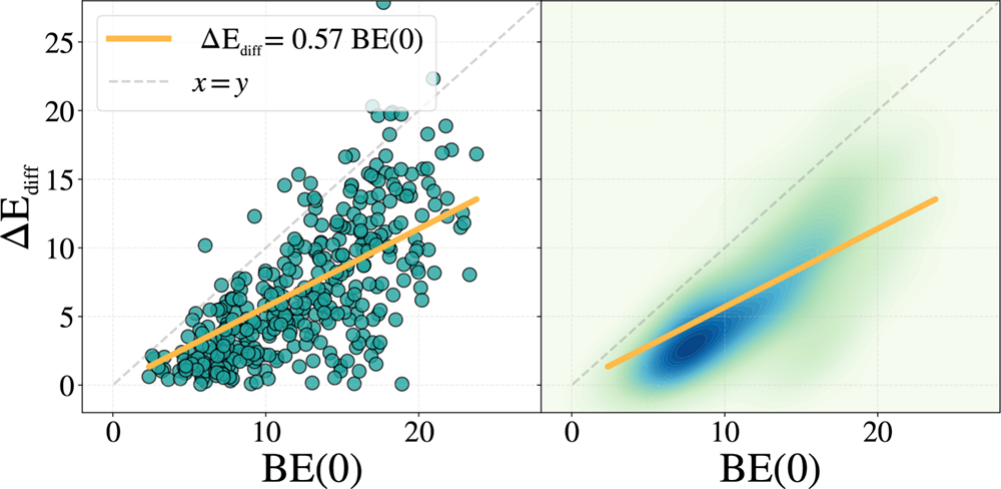
Correlation plot between Δ*E*_diff_ and the BE(0) using the values reported in Figure 3. On the left, the scattered plot is shown, while on the right, the same data are reported as kernel density estimate (KDE) to highlight the clusterization of the data. High concentration of points is identified by darker colors.

Here, Δ*E*_diff_ and BE(0) are computed following the scaling relations in Equation S7 and Equation S8 shown in the SI. The resulting average *f*-Ratio is approximately 0.6, spanning a broad range from 0.1 to 0.9. The average *f*-Ratio is in good agreement with the empirical estimate proposed by Garrod and Herbst.^8^ Nevertheless, it must be highlighted that the agreement is coincidental, as our value emerges from accurate calculations. Recently, Furuya et al. reported an experimental value of *f* = 0.4 for H_2_S.^48^ However, the adopted BE(0) was based on values from Penteado et al.,^87^ which in turn are derived from TPD measurements by Collings et al.^84^ using a linear scaling approach with water as the reference species. As previously discussed,^29,30^ such empirical correlations complicate direct comparison with computed data, and one-to-one correspondence should therefore be approached with caution.

In conjunction with the findings by Furuya et al.,^48^ which demonstrated the absence of a universal *f*-Ratio, it is crucial to highlight the significant variability associated with each diffusion barrier. As shown in Figure 5-right panel, the distribution is not uniform and the majority of diffusion barriers falls within the 5–10 kJ mol^–1^ interval. In Figure 6, we present the correlation between Δ*E*_diff_ with BE(0), where the latter has been averaged into bins of 5 kJ mol^–1^. Vertical bars in each bin represent the standard deviation, illustrating the spread of barrier heights across sampled diffusion paths. This approach mimics the manner in which BE distributions are typically implemented in astrochemical models and illustrates the range of possible diffusion barriers for each bin.^92^

**Figure 6:**
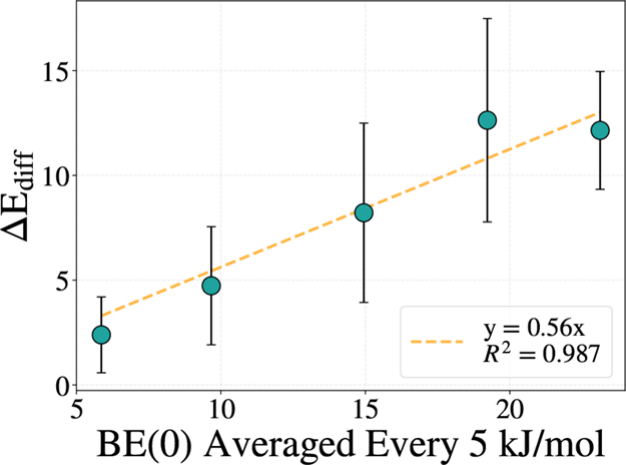
Correlation plot between Δ*E*_diff_ and BE(0) with standard deviation bars associated with each point. The orange dashed line is the linear fit with the intercept fixed to zero. The BE(0) values are binned every 5 kJ mol^–1^ and the diffusion barriers within each bin are averaged to obtain the points reported in the figure. Detailed data of this plot are reported in Table 2. All units are in kJ mol^–1^.

As evident in Figure 6, the diffusion barriers exhibit substantial variation around the mean BE(0) value, with the spread bars widening as BE(0) increases. In Table 2 the binned BE(0) values plotted in Figure 6 are reported. The mean *f*-Ratio varies from 0.4, at very low BE, to 0.66, when considering the high energy tail of the distribution.

**Table 2:** Binned Values Reported in Figure 6 Showing the Variability of the *f*-Ratio Associated with the Difference BE Range^a^

BE(0) range	BE(0) mean	Δ*E*_diff_ mean	*f*-ratio mean
2.4–7.4	5.86	2.39	0.409
7.4–12.4	9.67	4.73	0.483
12.4–17.4	14.95	8.22	0.549
17.4–22.4	19.22	12.63	0.658
22.4–27.4	23.13	12.15	0.524

a All values are reported in kJ mol^–1^ except for the f-ratio, which is dimensionless.

At temperatures typical of dense molecular clouds (10–20 K), thermal equilibrium is not achieved, and diffusion occurs over a heterogeneous energy landscape arising from the amorphous nature of the ice. Given that only low barriers are likely to be overcome under these conditions, a statistical representation of the *f*-Ratio is more appropriate. Consequently, the diffusion process should be represented not by a single mean *f*-Ratio, but by a subdistribution of values reflecting the variability of the underlying barriers. The binning adopted in our analysis (5 kJ mol^–1^) ensures that each subset contains a statistically meaningful number of diffusion events. This representation, in our opinion, offers a more physically realistic input for astrochemical models.

As similarly done in Zaverkin et al.,^59^ we can also compute the diffusion coefficient by adopting the classical Arrhenius expression reported in eqs 13 and 14. For Δ*E*_diff_ here we adopt the median value reported in Figure 4A, *a*_0_ can be obtained from the average distribution distance as reported in Figure 4C and is equal to 3.8 Å and finally, ν is the median value of the prefactor distribution, which is 3.2 × 10^12^ s^–1^.

With these values we obtain, *D*_0_^median^ = 3.6 × 10^–3^ cm^2^ s^–1^ and the diffusion coefficient *D* ^median^ = 2 × 10^–37^ cm^2^ s^–1^ at 10 K. Note that due to the very large distribution of diffusion barriers and distances, adopting this procedure to estimate the diffusion coefficient can be a crude approximation. Indeed, as shown by Zaverkin et al.,^59^ higher barriers have a larger influence on the residence time of the molecule, and therefore the explored range of barriers can largely deviate from the median value of the distribution reported in Figure 4A. For this reason, a more rigorous description has to be adopted. To this end, the kMC method offers a dynamic framework that explicitly incorporates the connectivity of adsorption sites and their associated diffusion barriers. By employing the computed distributions of BE and Δ*E*_diff_, the kMC simulation reproduces the stochastic evolution of diffusion events, providing insights into both the experimental observables and the behavior of H_2_S on icy grains under interstellar conditions.

### 3.2 Kinetic Monte Carlo

The aim of performing kMC simulations is 2-fold: to compute dynamically the diffusion coefficient of H_2_S and to evaluate how diffusion influences the BE(0) distribution and the corresponding TPD curve. In our previous work,^29^ based on static BE calculations, the simulated TPD peak differed from the experimental one by about 50–70 K. This motivated the present study, aimed at exploring whether or not molecular diffusion on ASW could contribute to such deviations. It is important to highlight that the kMC simulations presented here are not intended to reproduce a full TPD experiment but rather to isolate and quantify the influence of diffusion under controlled, isothermal, and low-coverage conditions.

Within this framework, several intrinsic differences with experimental TPD measurements must be noted. In the simulations, only a single H_2_S molecule interacts with the surface, whereas experimental spectra reflect mono- or submonolayer coverages where intermolecular interactions may occur, in particular upon diffusion. Moreover, real amorphous ice can undergo structural rearrangements or codesorption phenomena that are not captured in our modeling.

To explore the role of temperature under these simplified conditions, we performed kMC simulations at 15 fixed temperatures, from 10 to 80 K with 5 K of stepsize. This setup emulates the effect of a progressive temperature increase during TPD but replaces the continuous heating ramp with discrete isothermal holds (600 s each), allowing us to quantify how temperature affects the diffusion and desorption probabilities of a single H_2_S molecule on ASW. For each temperature, we started 10 simulations from every binding site of the distribution. This was done in order to ensure a proper convergence and sampling of the events space. It should be emphasized that we assumed the BE(0) and Δ*E*_diff_ values remaining constant within the temperature range explored during the kMC simulations (10–80 K) and do not significantly affect the final results. This assumption is supported by previous findings from Tinacci et al.^27^ (see their Figure D1), which demonstrated that temperature effects become significant only above 100 K. Rates were calculated according to eqs 5 and 9. Given that tunneling effects on the diffusion coefficient are already negligible for lighter atoms such as nitrogen,^59^ we have omitted these effects here, considering the greater mass of the sulfur atom.

Figure 7A shows the comparison between the BE(0) distribution used in the previous work by Bariosco et al.^29^ and the one adopted in this work by applying a scaling factor on DFT results in order to reach DLPNO–CCSD­(T) accuracy (see SI Section 2 for details). As it can be seen, the two distributions vary by ∼5 kJ mol^–1^ in the mean value. With respect to the TPD published in Bariosco et al.,^29^ the new temperature peak (*T*_peak_) is shifted up by ∼10 K, centered at ∼40 K. Given the small deviation and the substantial savings in computational time, we used the scaled data to perform the kMC simulations.

**Figure 7:**
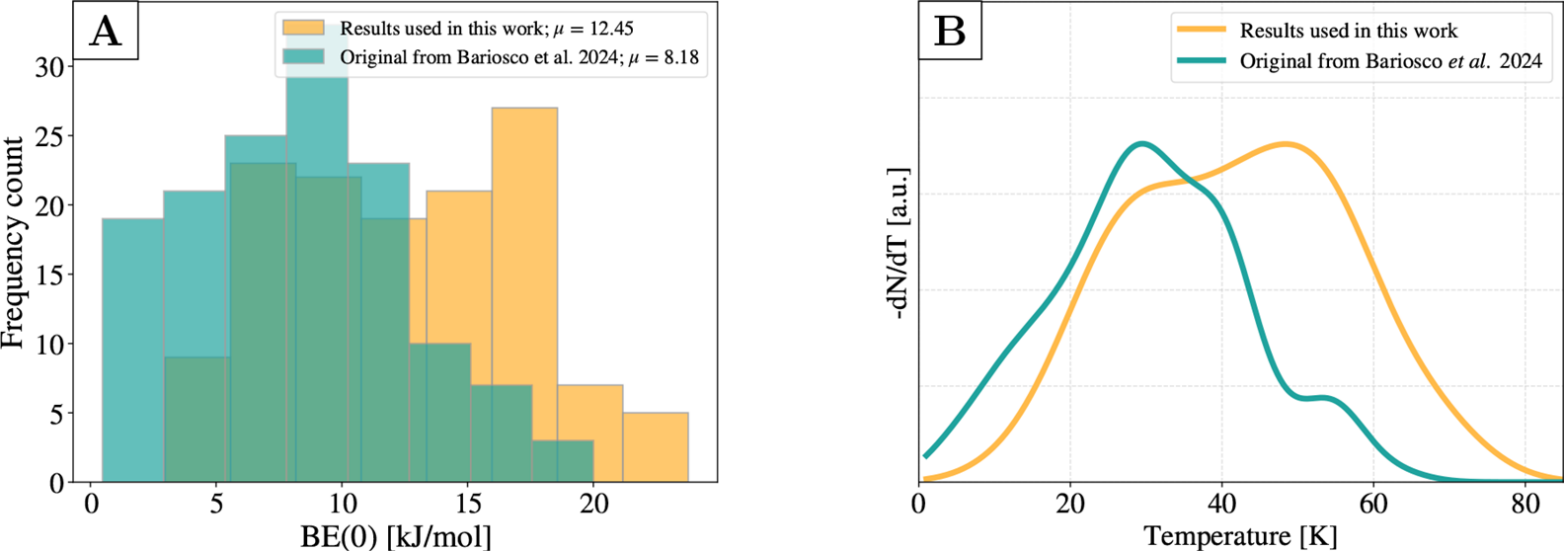
Comparison between the data set originally used in the work by Bariosco et al.^29^ and the data used in this work between (A) binding energy distributions and (B) simulated TPD curve. The heating rate is fixed at 4 K min^–1^, while the prefactor is evaluated through Tait et al.'s formula.^70^ The number of bins and their widths are obtained following the Freedman Diaconis estimator.^83^

To investigate the impact of diffusion on desorption, we reconstructed an approximate TPD profile from the kMC simulations. During each simulation, whenever a desorption event occurs, the simulation is interrupted and the binding site from which desorption occurs is recorded. By collecting these events across temperatures, we generated the desorption profile shown in Figure 8, where the number of desorption events at each temperature is plotted as a histogram. This profile is subsequently fitted with a sigmoidal function using the following parameters:

$$N\left(T\right)=\frac{A}{1+\exp\left[-z\left(T-T_{i}\right)\right]}$$ 15

where *N*(*T*) is the number of desorption events at the temperature (*T*), *A* = 1357.2 is its maximum value, *z* = 0.119 K^–1^ is the growth rate per Kelvin degree of the sigmoidal curve and *T*_*i*_ = 44 K is the inflection point.

**Figure 8:**
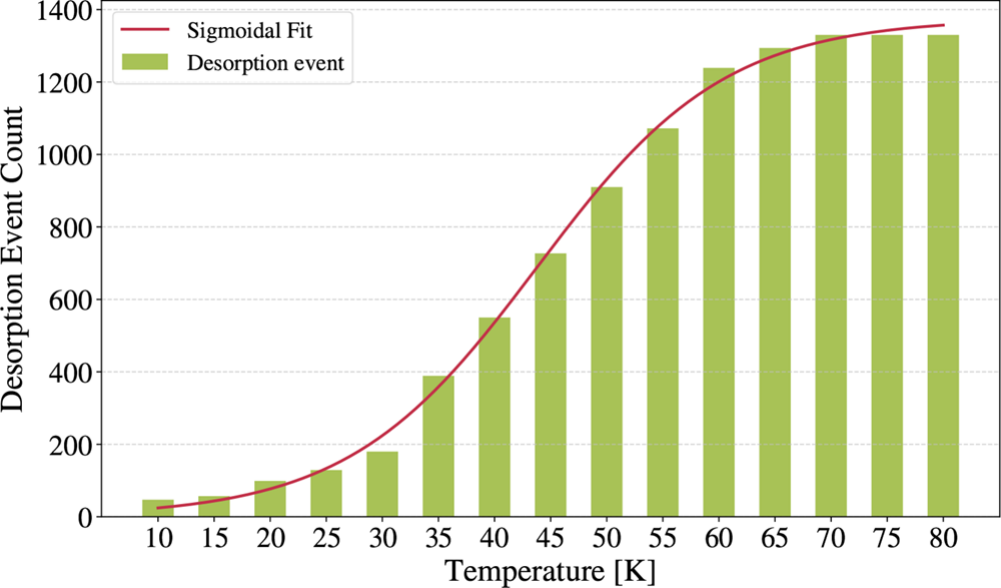
Desorption event counts obtained from the kMC results. The histogram plot is fitted with a sigmoidal function (see eq 15).

As shown in Figure 8, the maximum number of desorption events is reached already at 70 K, while *T*_*i*_ of the sigmoidal curve is observed around 45 K. The desorption rate was obtained by analytically differentiating the sigmoidal profile *N*(*T*) with respect to temperature:

$$\frac{dN(T)}{dT}=\frac{Az\;\exp(-z(T-T_{i}))}{(1+\exp(-z(T-T_{i})){)}^{2}}$$ 16

providing a desorption rate analogous in shape to a TPD curve, enabling qualitative comparison with the Polanyi–Wigner result in Figure 9. As it can be noted, within the present isothermal framework, the effect of diffusion on the position of the *T*_peak_ is modest. While the original TPD peaked at 40 K, the reconstructed TPD shows *T*_peak_ around 45 K.

**Figure 9:**
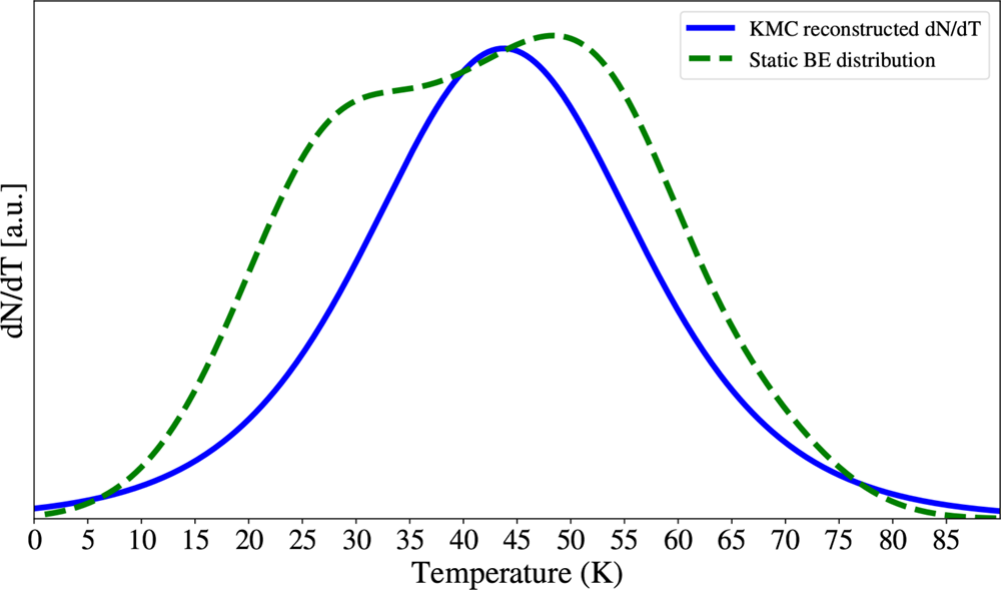
Comparison between two TPD curves. The dashed green curve is the TPD spectrum obtained through the numerical evaluation of the Polanyi–Wigner equation adopting Tait et al.'s prefactor^70^ and the BE distribution illustrated in Figure 7A (orange bins) and already shown in Figure 7B (orange curve). The blue solid line is the TPD spectrum reconstructed by derivation of the sigmoidal fit reported in Figure 8 using eq 16. Reproduced from Bariosco et al.^29^ Copyright 2024, MNRAS.

Although the relative likelihood of diffusion exceeds that of desorption (see Figure 5), the limited density of high binding energy sites hinders the extent to which diffusion can alter the overall desorption profile. Under experimental conditions, the outcome could differ: a continuous heating ramp and thermally induced structural rearrangements of amorphous ice may locally reshape the adsorption landscape, effectively converting shallow sites into deeper ones without requiring long-range molecular motion.

Our kMC results indicate that under submonolayer conditions, H_2_S diffusion does not significantly affect the position of the TPD peak, and thus has a negligible impact on the evaluation of the BE distribution. Furthermore, diffusion could become important in the formation of small islands or aggregates on the ASW surface. Indeed, as observed for other molecules,^93^ even in the submonolayer regime, the initial heating ramp in TPD experiments can promote the formation of islands, driven by molecular diffusivity. In the case of weakly bound species like H_2_S, the formation of such islands can alter the apparent binding energy by introducing lateral interactions among adsorbates. These cooperative effects can increase the final BE value.

In Figure 10, we present the temperature-dependent diffusion coefficients for the H_2_S molecule on amorphous water ice, along with the corresponding Arrhenius fit (see eq 13). Diffusion coefficients were fitted until 25 K, where equilibrium conditions are fully reached by our simulations.^59^ The analysis of the fit yields a diffusion coefficient prefactor of *D*_0_ = 6.3 ± 2.0 × 10^–3^ cm^2^ s^–1^ and an effective diffusion barrier of 8.6 ± 0.2 kJ mol^–1^. Comparing this with the static analysis based on eq 14 in Section 3.1, we find that the *D*_0_ increases with respect to the averaged value (*D*_0_^median^ = 3.6 × 10^–3^ cm^2^ s^–1^), as well as the median diffusion barrier (5.4 ± 3.5 kJ mol^–1^), which still remains consistent with the experimental results of Furuya et al. (7.2 kJ mol^–1^).^48^ This suggests that a model incorporating desorption events yields a lower mobility compared to estimations based solely on QM calculations.

**Figure 10:**
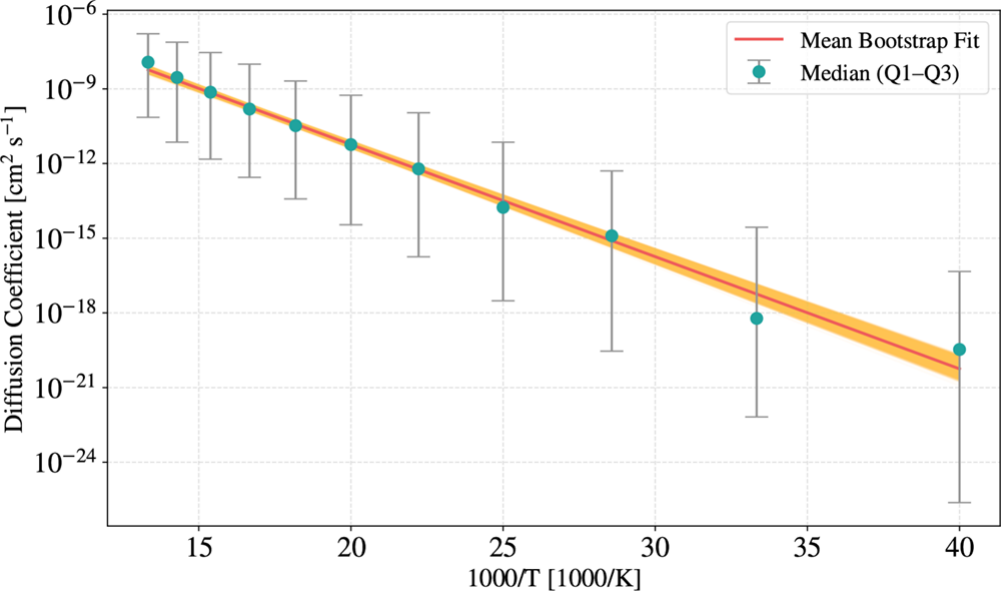
Temperature dependence of diffusion coefficients for H_2_S on amorphous water ice from 75 to 25 K. Circles represent the median diffusion coefficient at each temperature with error bars showing the interquartile range (Q1–Q3). The red line shows the mean Arrhenius fit obtained from 20,000 bootstrap iterations. The orange shaded region represents the distribution of all 20,000 bootstrap fits, visualizing the uncertainty propagation from the data to the fitted parameters.

In Table 1, we summarize the *D*_0_ reported in the literature for various species. The prefactor *D*_0_ is related to the attempt frequency ν_diff_ via:

$$D_{0}=\frac{1}{2d}\nu_{\mathrm{diff}}\lambda^{2}$$ 17

where *d* is the dimensionality of the system and λ is the hopping length. Computational studies, including the present work, typically yield *D*_0_ values in the range of 10^–2^–10^–3^ cm^2^ s^–1^, whereas experimental results span a broader and generally lower range, from 10^–4^ to 10^–8^ cm^2^ s^–1^. This discrepancy is largely attributable to the different assumptions used to estimate the prefactor ν_diff_. Indeed, computational studies typically assume ν_diff_ values on the order of 10^12^ s^–1^, reflecting the characteristic soft vibrational modes (50–250 cm^–1^) along the reaction coordinate in weakly bound systems. Notably, the experimental study of CO_2_ diffusion by Kouchi et al., which assumed a fixed ν_diff_, reported a *D*_0_ value more consistent with computational predictions. Given the strong sensitivity of *D*_0_ and *E*_diff_ to the choice of ν_diff_, further investigations are needed to better quantify its influence on molecular mobility on ASW.

Using the parameters from the Arrhenius fit reported in Figure 10, we extrapolate the diffusion coefficient of H_2_S at 10 K to be *D* = 2 ± 5 × 10^–47^ cm^2^ s^–1^, a value substantially lower than that obtained from the approximate parameters in eq 14, which yields *D* ^median^ = 2 × 10^–31^ cm^2^ s^–1^. Assuming diffusion on a two-dimensional surface, the MSD is given by ⟨*r* ^2^⟩ = 4*Dt*. Accordingly, the time required for an H_2_S molecule to scan a distance of 1 μm on a water-dominated icy dust grain at 10 K is approximately *t* ∼ 10^38^ s (∼10^30^ years) using the fitted *D*, and *t* ∼ 10^21^ s (∼10^14^ years) using the averaged *D* ^median^. At 20 K, the corresponding time reduces to *t* ∼ 10^16^ s (∼10^8^ years) when using the *D* value derived from the linear fit in Figure 10. These results strongly suggest that thermally activated diffusion of H_2_S, hence Langmuir–Hinshelwood reactions involving this species, are highly improbable under typical dense cloud conditions (*T* ∼ 10–20 K) and existence time of the molecular clouds (∼10^6^ years).

Finally, it is pertinent to consider at which temperature thermal diffusion of H_2_S on ASW becomes astrophysically relevant. Defining an absolute threshold value of the diffusion coefficient to assess surface reactivity remains a nontrivial task; nevertheless, a useful point of comparison can be drawn from the case of atomic hydrogen diffusion on ASW. Among the numerous studies dedicated to this topic,^33,56,58,94,95^ the recent work by Asgeirsson et al.^56^ is particularly relevant due to methodological similarities with the present study. They report a diffusion coefficient of *D*_H_ = 5.80 × 10^–11^ cm^2^ s^–1^ at 25 K. In order to achieve a comparable diffusion coefficient for H_2_S would require raising the temperature to approximately 55 K.

## 4 Conclusions

In this work, we presented a robust and automated atomistic modeling approach based on QM calculations combined with kinetic Monte Carlo simulations to characterize molecular diffusion on amorphous interstellar ices. Building upon a previously reported BE distribution of H_2_S on ASW, we constructed a high-resolution diffusive network by identifying 141 adsorption sites and more than 270 transition states, benchmarked against DLPNO–CCSD­(T) for chemical accuracy. Our analysis revealed a wide distribution of diffusion barriers ranging from 0.1 to 27 kJ mol^–1^, with a median of 5.4 kJ mol^–1^, and a significant number of quasi-barrierless pathways. These findings underscore the complexity and flatness of the PES in weakly bound systems, and highlight the importance of resolving sub-kJ mol^–1^ energy differences with high-level methods.

Our kMC simulations explicitly include desorption and were run at 15 fixed temperatures (10–80 K, 5 K stepsize; 600 s per isotherm) under single-molecule, submonolayer conditions. An approximate TPD profile reconstructed from desorption events shows only a modest shift of the peak temperature relative to the static Polanyi–Wigner result (∼45 vs ∼40 K), indicating that diffusion exerts little influence on the peak position at low coverage within the present isothermal protocol. Nonetheless, during the initial stages of experimental heating, limited diffusion may promote surface clustering of weakly bound species, enhancing lateral interactions and effectively increasing the apparent binding energy. Incorporating such cooperative effects into future kMC frameworks would improve the realism of TPD modeling.

A central result is the absence of a universal scaling between BE(0) and Δ*E*_diff_. The ratio *f* = Δ*E*_diff_/BE spans ∼0.1 to 0.9, with binned mean values varying from ∼0.40 at low BE to ∼0.66 in the high-energy tail. This variability suggests that adopting a fixed fraction of BE to estimate diffusion barriers introduces substantial uncertainties and fails to capture the inherent heterogeneity of ASW surfaces. Instead, we recommend incorporating distributions of both BE and diffusion barriers in astrochemical models rather than single-value parametrizations.

From Arrhenius analysis of kMC-derived diffusion coefficients, we obtain *D*_0_ = 6.3 ± 2.0 × 10^–3^ cm^2^ s^–1^ and an effective barrier of 8.6 ± 0.2 kJ mol^–1^. Extrapolation to 10 K yields *D* ≈ (2 ± 5) × 10^–47^ cm^2^ s^–1^, effectively ruling out thermally driven H_2_S surface mobility, thus Langmuir–Hinshelwood mechanisms, in dense clouds (10–20 K).

In summary, the central innovation of this work is the development of a broadly applicable framework for simulating the statistically significant variety of diffusion pathways of a species adsorbed on icy surfaces, with high accuracy and computational efficiency. This methodology enables the generation of realistic diffusion barrier distributions for interstellar molecules, representing a key advancement with important implications for astrochemical modeling.

## Supplementary Material



## Data Availability

All Cartesian coordinates, input and output files, and the full energetics data set are deposited at the following link: https://zenodo.org/records/17832299. The kMC code used in this work along with all the data set containing the energetic information is freely available at the following link https://github.com/Viba97/kMC4D.git. To efficiently handle the large data set of BE samples and Diffusion barriers, we have developed and made publicly available a Web site based on the molecule hyperactive JSmol plugin (https://jmol.sourceforge.net). This electronic version includes the 133 BE sites and the 370 diffusion pathways at the ONIOM­(B97–3c:xTB-GFN2) level and can be explored interactively on GitHub at this link https://viba97.github.io/H2S_diffusion_3D_visualization/.

## Notes

The construction of the diffusive network, including the relevant graph theory concepts employed (Section 1); the energetic benchmarks for the discussed results (Section 2), an analysis of the effect of the model zone on the BE values (Section 3); the relationship between the diffusion barrier and the energy difference between minima (Section 4); and the convergence of the KMC simulations (Section 5) (PDF)
