# Supplementary material for: A Fully ab Initio Kinetic Monte Carlo Approach for Modeling Adsorption and Diffusion in Interstellar Icy Grain Mantles: The Case of H2S
Source: ACS Earth Space Chem. 2025 Dec 18;10(1):66–79. doi: 10.1021/acsearthspacechem.5c00208 (PMC12814774; doi:10.1021/acsearthspacechem.5c00208)
Supplement: Supplementary file 1 [file sp5c00208_si_001.pdf]

Supporting Information
A fully *ab initio* kinetic Monte Carlo approach for modelling adsorption and diffusion in interstellar icy grain mantles: the case of H_2S

Vittorio Bariosco,*,†,‡ Stefano Pantaleone,‡ Cecilia Ceccarelli,¶ Piero Ugliengo,‡ and Albert Rimola*,†,§

†*Departament de Quimica, 08193 Bellaterra, Catalonia, Spain*

‡*Dipartimento di Chimica, via P. Giuria 7, I-10125 Torino, Italy*

¶*Institut de Planetologie et d'Astrophysique de Grenoble (IPAG), F-38000 Grenoble, France*

§*Accademia delle Scienze di Torino, Via Maria Vittoria, 3, 10123 Torino, Italy*

E-mail: vittorio.bariosco@uab.cat; albert.rimola@uab.cat

1 Diffusive Network

We adopted the Closeness Centrality C(u) concept:

$$\mathrm{C(u)} = \frac{\mathrm{n} - 1}{\sum_{\mathrm{u} \neq \mathrm{v}} \mathrm{d(u, v)}} \tag{S1}$$

where n is the number of reachable nodes from u, and d(u, v) represents the geodesic distance between two nodes on the grid, (u, v).[1] This distance, also known as the shortest path connecting two points on the grid, measures the minimum number of steps required to traverse between them.[2] C(u) quantifies how quickly a node can access all other nodes in a network. In the context of diffusion, a node with a high C(u) value is able to interact with other nodes more efficiently, as it is generally closer to all other nodes in terms of the shortest path distance. To provide a clearer understanding of this concept, Figure S1 presents a simplified example of two possible grid configurations. In the grid shown in Figure S1-A, the shortest path connecting node "a" to node "d" is 2, as they are indirectly connected, while the one connecting "a" to "b" or "c" is equal to 1. The same is true for all other nodes in the 2x2 grid. Consequently, considering that d(a,b)=d(a,c)=1 and d(a,d)=2, the average $\bar{\mathrm{C}}$(u) is calculated as follows:

$$\begin{aligned} \mathrm{C(a)} &= \frac{3}{(\mathrm{d(a,b)}) + (\mathrm{d(a,c)}) + (\mathrm{d(a,d)})} = 0.75 \\ \bar{\mathrm{C}}(\mathrm{a,b,c,d}) &= \frac{\mathrm{C(a)} + \mathrm{C(b)} + \mathrm{C(c)} + \mathrm{C(d)}}{4} = 0.75 \end{aligned} \tag{S2}$$

due to the fact that all the nodes are connected in the same manner. Nevertheless, if we try to increase the connectivity of our grid (see Figure S1-B), $\bar{\mathrm{C}}$(u) becomes:

$$\bar{\mathrm{C}}(\mathrm{a,b,c,d}) = \frac{1 + \frac{3}{4} + \frac{3}{4} + 1}{4} = 0.875 \tag{S3}$$

demonstrating indeed that the nodes can interact in a faster way between each other.

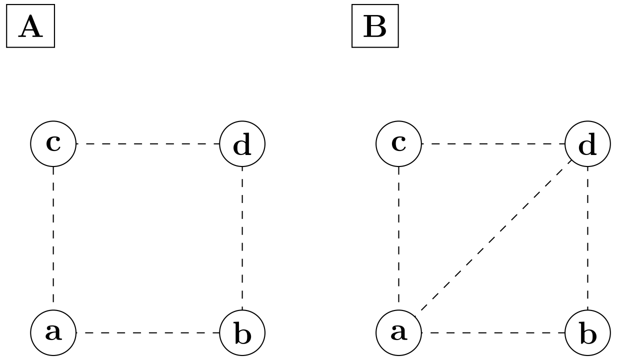

Figure S1: Sketched examples of two differently connected grids. (A) 2x2 grid with 4 nodes (circle markers) and 4 edges (dashed lines). (B) 2x2 grid with 4 nodes and 5 edges.

Given that the minima identified on the grain can sometimes be disconnected or sparsely connected, a slight modification of Equation S1 must be adopted. Specifically, the Wasserman-Faust function provides an improvement over the standard closeness centrality formula.[2] In traditional closeness centrality, nodes in small disconnected components can receive disproportionately high scores because their average distance to reachable nodes is relatively short, even though they are isolated from the rest of the graph. The Wasserman-Faust formula addresses this by scaling the centrality score according to the size of the entire graph, including unreachable nodes. This adjustment ensures that disconnected nodes receive lower centrality values, accurately reflecting their limited access to the overall network. The modified expression is defined as:

$$\mathrm{C_{WF}(u)} = \frac{\mathrm{n}-1}{\mathrm{N}-1}\frac{\mathrm{n}-1}{\sum_{\mathrm{u}\neq\mathrm{v}} \mathrm{d(u,v)}} \tag{S4}$$

where N is the total number of nodes in the grid.

As the grid size increases, the unnormalized closeness centrality ($\mathrm{C_{WF}(u)}$) becomes less suitable for comparing nodes across grids with varying levels of connectivity. This is because the raw values of $\mathrm{C_{WF}(u)}$ can be strongly influenced by the overall size and structure of the grid. To address this limitation, we apply a normalization procedure, defining the normalized closeness centrality as:

$$C^{N}_{WF}(u) = \frac{C_{WF}(u)}{C^{max}_{WF}} \tag{S5}$$

where C^{max}_{WF} is the maximum among all the $C_{WF}(u)$ values of the grid.

The diffusive grids, generated following the procedure detailed in Section 2.2, are presented in Figure S2. To visualize the spatial distribution of points on a spherical surface in two dimensions, a spherical-to-planar mapping was applied. In this transformation, spherical coordinates (θ, ϕ) (with θ as the polar angle and ϕ as the azimuthal angle) were projected onto Cartesian coordinates according to:

$$x = 2\sin\theta\cos\phi, \quad y = 2\sin\theta\sin\phi \tag{S6}$$

Figure S2-A shows the initial grid, which consists of 820 diffusive paths connecting 141 binding sites. As discussed, the normalized closeness centrality ($C^{N}_{WF}(u)$) serves as a valuable descriptor for assessing the interconnection within a grid of points and can be used for comparative analysis across different grids. In this study, we utilize this metric to gain insights into the mobility of individual points. The average normalized closeness centrality in Figure S2-A is approximately 0.8, indicating that, on average, the mobility of the nodes is 80% of the most mobile node in the grid. Given the amorphous nature of the grain and the uneven distribution of binding sites, this value reflects a reasonably well-connected network. However, the grid depicted in Figure S2-A represents an idealized case, where all initially identified paths correspond to true TS. A more realistic scenario is shown in Figure S2-B, where the diffusive paths have been filtered according to the procedure outlined in Section 2.2. In this case, the average ($C^{N}_{WF}(u)$) decreases by approximately 0.1%, which remains acceptable considering the total number of edges (273 computed TSs). Additionally, the normalized closeness centrality ($C^{N}_{WF}(u)$) provides crucial information about isolated nodes that are unable to diffuse to other binding sites. A low $C^{N}_{WF}(u)$ suggests the presence of such isolated binding sites, which can impact the residence time in kMC simulations. In the final

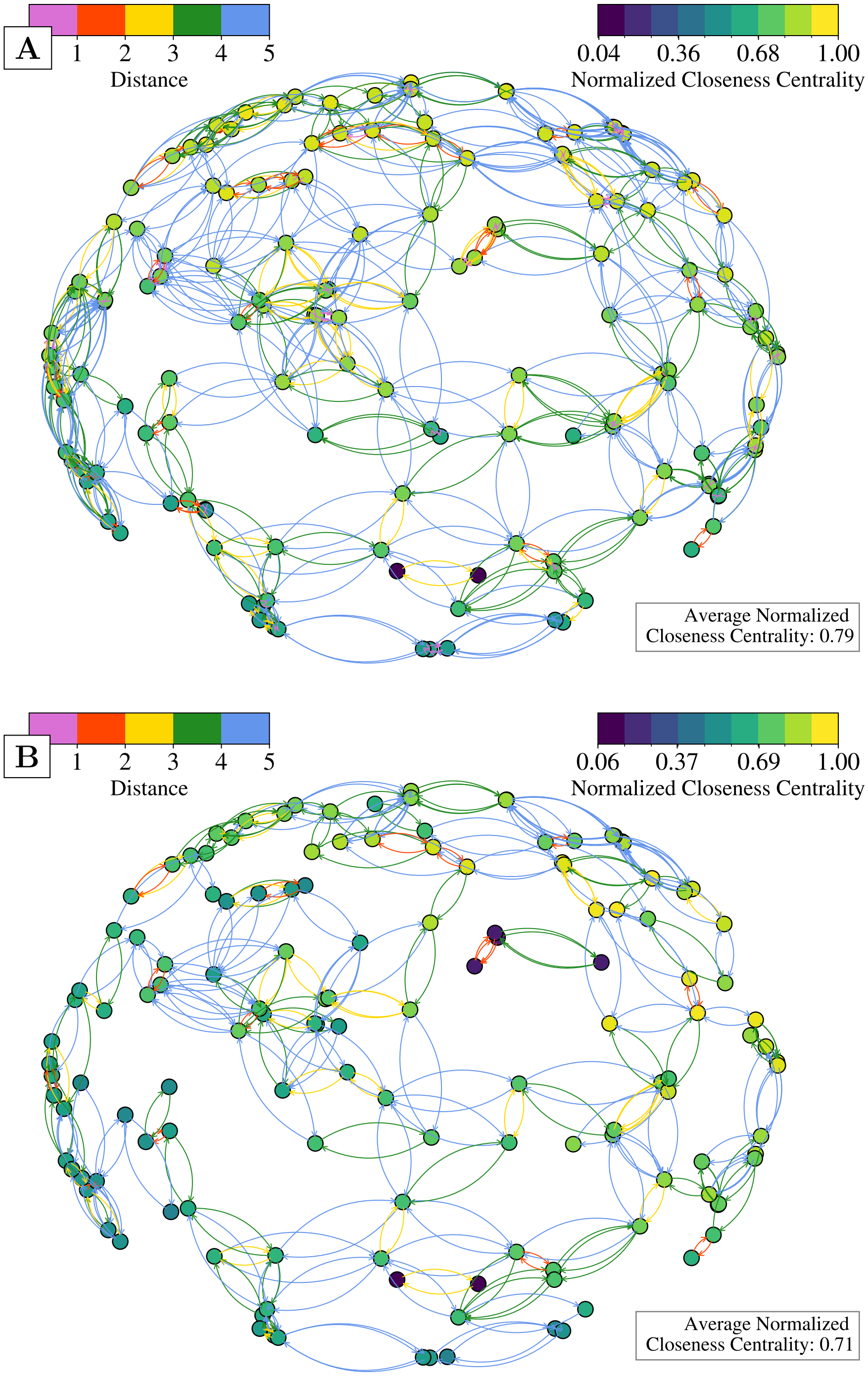

Figure S2: Diffusive network composed by 141 minima (points) for the (A) unpruned (820 edges) and (B) pruned (546 edges) grids, according to the filtering criteria outlined in Section 2.2. The color map on the left represents the distance between the nodes (in Å), the one on the right reports the normalized closeness centrality value ($C^{N}_{WF}(u)$). The darkest points are the one less connected to the grid.

grid shown in Figure S2-B, two main clusters of isolated binding sites (highlighted in dark purple) are identified. These six sites are subsequently excluded from the diffusive network.

2 ΔE_{diff} and BE benchmark B97-3c vs DLPNO-CCSD(T)

A recent study demonstrated that the B97-3c method is sufficiently accurate for describing $H_2S \cdots H_2O$ interactions, which, however, are usually overestimated.[3] This behaviour becomes particularly critical when the energies involved are close or below the chemical accuracy threshold (≤5 kJ mol^{-1}), as it usually happens for diffusion processes of weakly bound systems.[4–6] To enhance our energy calculations accuracy, we decided to use the more accurate DLPNO-CCSD(T) method. However, due to the large number of required calculations and the increased Model zone (as shown in Figure 2), performing these calculations would be computationally unfeasible.

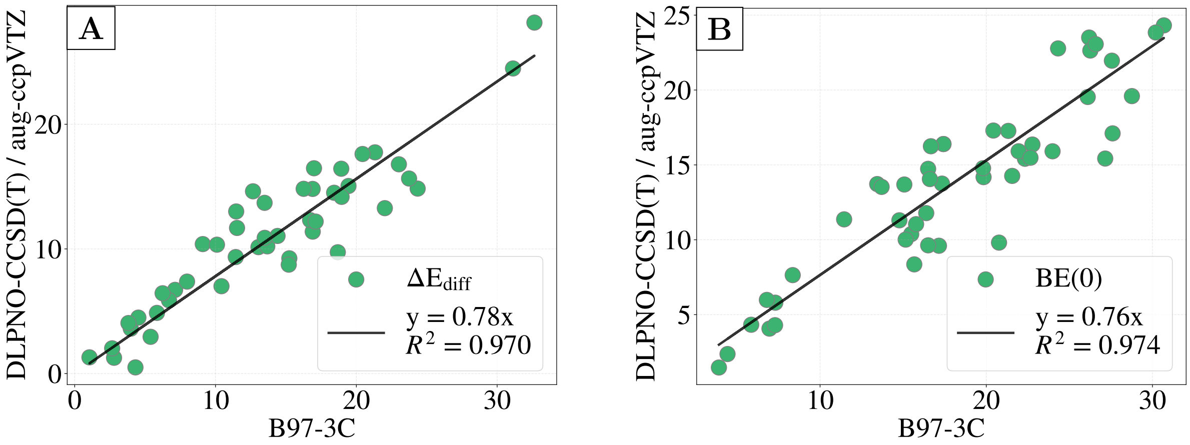

Figure S3: Correlation plots between DLPNO-CCSD(T)/aug-ccPVTZ and B97-3c functional. (A) Diffusion barrier ΔE_{diff} computed with the two previously mentioned methods. (B) Binding energy corrected for the ZPE (BE(0)) computed with the two previously mentioned methods. All the values are reported in kJ mol^{-1}.

To address this, we selected 28 diffusion paths and optimized the MEP. For 18 of these paths, we successfully found the TS directly after performing the CI-NEB calculations. For 6 additional paths, we located the TS through further TS optimizations. However, 4 paths did not converge to a valid TS and were discarded from the dataset. After applying the

ZPE correction, four cases exhibited negative barriers and were thus excluded from the benchmark dataset. Figure S3-A presents the correlation plot of the ΔE_{diff} between B97-3c and DLPNO-CCSD(T) methods for the 18 diffusive paths considered. It should be noted that the total number of data points is 36, as each diffusive path includes barriers in both the forward (reactant to product) and reverse (product to reactant) directions (see Equation 4 in the manuscript). As reported in Figure S3-A, the ΔE_{diff} values computed with the two methods correlate reasonably well, with a scaling factor of 0.78. For this reason, and to save significant computational time, we applied a scaling factor to all the B97-3c ΔE_{diff} values using the relation:

$$\Delta E_{diff}(\text{DLPNO-CCSD(T)}) = 0.78 * \Delta E_{diff}(\text{B97-3c}) \tag{S7}$$

With the same objective, we analyzed the correlation for the binding energy corrected for the ZPE (BE(0)). In Figure S3-B, the same correlation plot but for the BE(0) is illustrated. The quality of the correlation slightly improves (R^2=0.974), with a gradient of 0.76. Also for this case we apply this factor to obtain the final BE(0) value, following this equation:

$$\text{BE(0)(DLPNO-CCSD(T))} = 0.76 * \text{BE(0)(B97-3C)} \tag{S8}$$

3 Model zone convergence

Although the ONIOM method is an efficient methodology to combine accuracy and computational cost, the selection of the Model zone is a critical part to describe the system correctly. Tinacci *et al.*[7] demonstrated that BEs obtained using the ONIOM procedure are well-converged values, and in our previous work on H_2S, we confirmed similar findings.[3] However, it is important to note that, while H_2S BEs are considered converged, increasing the Model zone could still introduce random variations in the BEs, depending on the number and arrangement of the water molecules included in the extended model.

We verified this effect by correlating the BE value before and after the re-optimization of the Model zone (see Step iv) in Section 2.2). In Figure S4, illustrates the BE computed before (BE_{PRE}) and after (BE_{POST}) the re-optimization at B97-3c level, in the color bar is represented the difference in the number of H_2O molecules after the new Model zone is defined. The correlation between the two values is tight (see Figure S4) corroborating what already has been published. However, as anticipated some values strongly deviate from the fit, describing in most of the cases a higher BE value when the zone is increased.

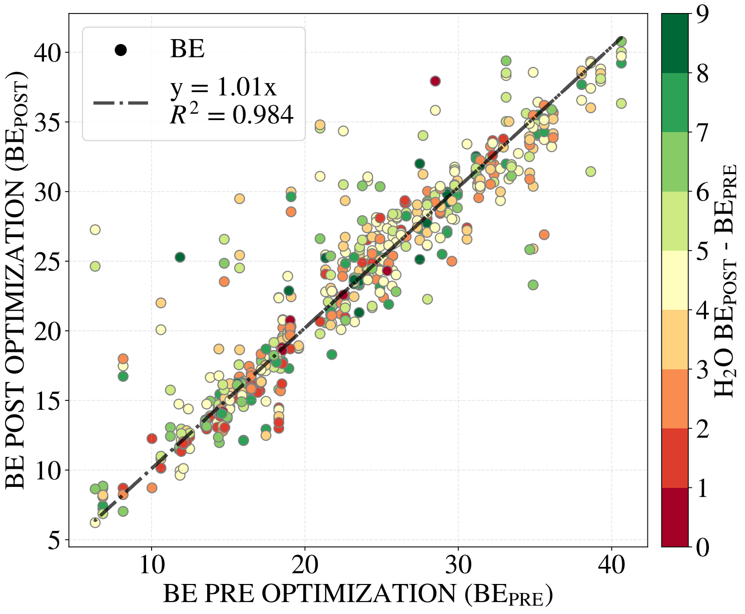

Figure S4: Correlation plot between the BE computed before (BE_{PRE}) and after (BE_{POST}) the new definition of the Model zone (see Step iv) in Section 2.2). In the color map is represented the difference between the number of water molecules included in the Model zone before and after the New Model zone. BEs were not corrected for ZPE. All the values are reported in kJ mol^{-1}.

4 ΔE_{diff} vs ΔBE

Another parameter commonly discussed in the literature is the correlation between the diffusion barrier connecting two adsorption minima and the corresponding energy difference between them.[8,9] This relationship is shown in Figure S5. No clear correlation emerges from

our data, in agreement with the findings of Cuppen *et al.*[9] for CO physisorbed on amorphous solid water (ASW). Overall, the considerations previously discussed for the uncertainty associated with the f-ratio are equally applicable here.

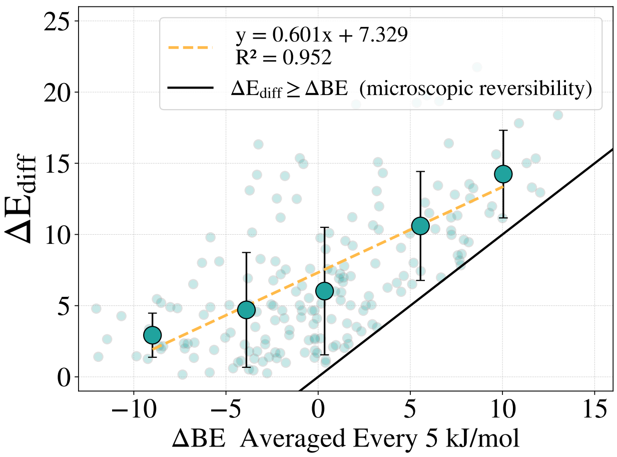

Figure S5: Correlation plot between ΔE_{diff} and $\Delta BE(0)$ with the standard deviation bar associated with each point. The orange dashed line is the linear fit over the mean value (larger points). The BE(0) is collected every 5 kJ mol^{-1} and averaged to obtain the points reported in the Figure. The microscopic reversibility condition is shown with the black x=y function.

5 kMC convergence

Figure S6 presents D and mean squared displacement (MSD) obtained from kMC trajectories at several temperatures starting from one adsorption site. The left panel shows the evolution of D with iteration number, while the right panel reports the corresponding MSD as a function of time, illustrating the overall diffusive behaviour of the system. These plots can be summarized:

- 10 K: The molecule rapidly diffuses between two binding sites but remains confined within this local region. The desorption probability is negligible, and no desorption events are observed in any of the independent trajectories. All simulations terminate upon reaching the maximum allowed simulation time (600 s).

- 20 K: H_2S initially explores two binding sites distinct from its starting position, then returns to the initial site at approximately 10^{-3}–10^{-1} s. This transition requires an average time step of 5 s due to the high energy barrier crossed at this low temperature. For both the 10 K and 20 K simulations, convergence of the diffusion coefficient D is achieved rapidly, as the molecule remains trapped in deep binding sites separated by high diffusion barriers, permitting only limited hopping events.

- 30 K: The initial dynamics resemble those observed at 20 K. Desorption events become increasingly probable, with two occurrences at approximately 10^{-1} s. At this temperature, the system overcomes two additional energy barriers, accessing distinct binding sites characterized by MSD values of ~30 $Å^2$ and ~60 $Å^2$. Overcoming these barriers requires time steps on the order of 2×10^5 s, which significantly exceeds typical experimental timescales.

- 50–70 K: Desorption becomes the dominant process as the system crosses multiple energy barriers. The stochastic nature of the kMC algorithm is evident from the variation in iteration number and time at which desorption occurs across different trajectories. The kMC desorption probability is accurately reproduced, as demonstrated by the observation that, following surface diffusion, desorption consistently occurs from the same lowest-energy binding site.

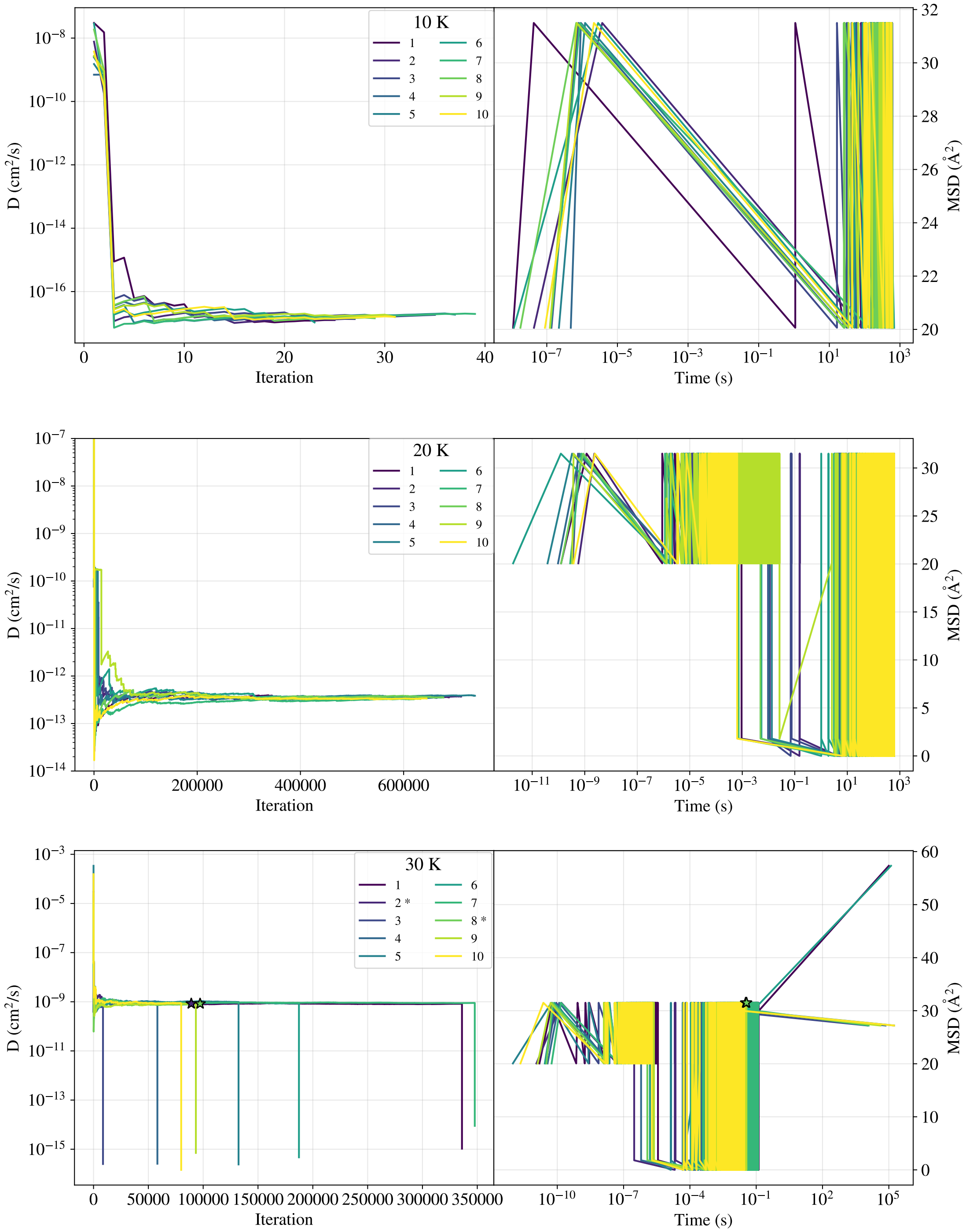

Figure S6: Evolution of the cumulative diffusion coefficient (left) and mean squared displacement (right) for the ensemble of kMC trajectories starting from site 369 at different temperatures. Each curve corresponds to an independent simulation run, color-coded for clarity. A star indicates the iteration or time step at which desorption occurred, marking the end of the diffusion process for that trajectory.

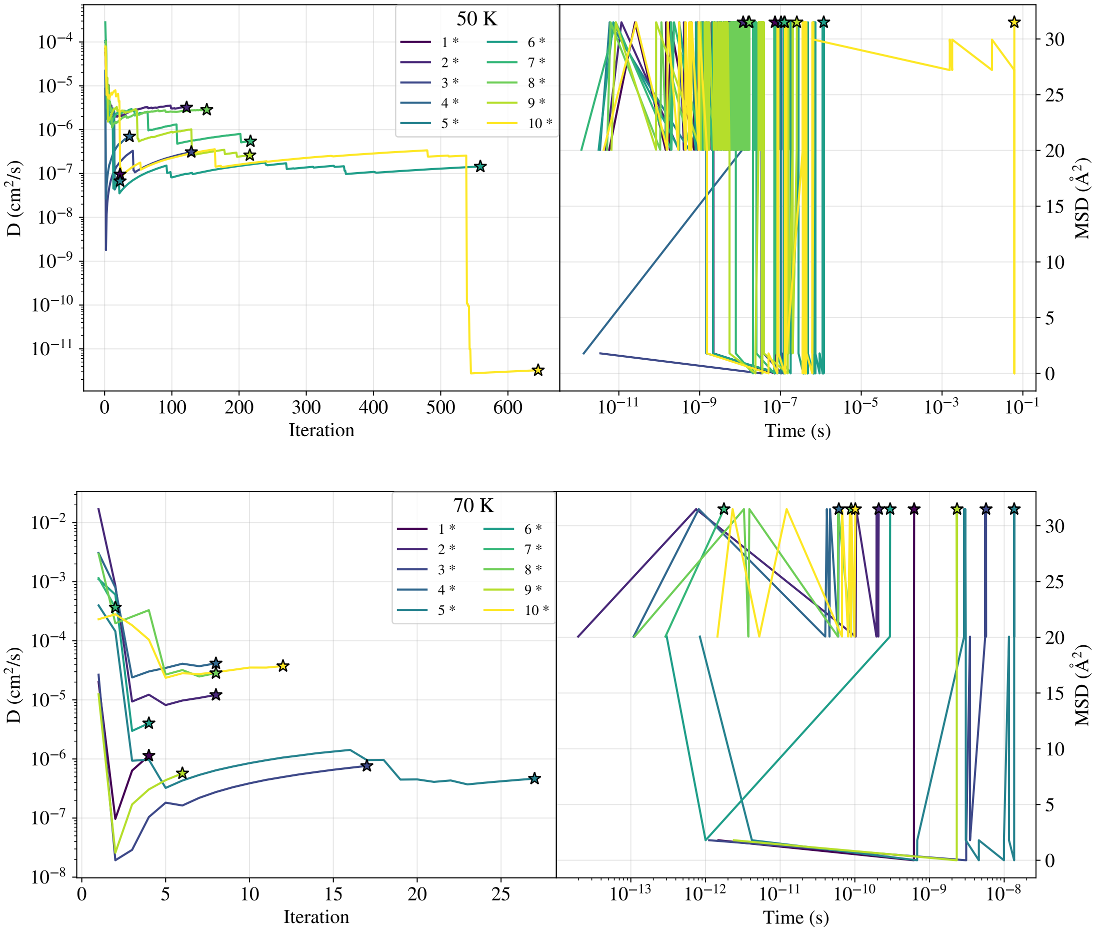

Figure S6: Figure S6 (continued).

References

(1) Freeman, L. Centrality in social networks conceptual clarification. *Social Networks* **1978**, *1*, 215–239.

(2) Wasserman, S. Social network analysis: Methods and applications. *The Press Syndicate of the University of Cambridge* **1994**,

(3) Bariosco, V.; Pantaleone, S.; Ceccarelli, C.; Rimola, A.; Balucani, N.; Corno, M.; Ugliengo, P. The binding energy distribution of H_2S: why it is not the major sulphur reservoir of the interstellar ices. *Monthly Notices of the Royal Astronomical Society* **2024**, *531*, 1371–1384.

(4) Karssemeijer, L.; Cuppen, H. Diffusion-desorption ratio of adsorbed CO and CO2 on water ice. *Astronomy & Astrophysics* **2014**, *569*, A107.

(5) Molpeceres, G.; Zaverkin, V.; Kästner, J. Neural-network assisted study of nitrogen atom dynamics on amorphous solid water–I. adsorption and desorption. *Monthly Notices of the Royal Astronomical Society* **2020**, *499*, 1373–1384.

(6) Zaverkin, V.; Molpeceres, G.; Kästner, J. Neural-network assisted study of nitrogen atom dynamics on amorphous solid water–II. Diffusion. *Monthly Notices of the Royal Astronomical Society* **2022**, *510*, 3063–3070.

(7) Tinacci, L.; Germain, A.; Pantaleone, S.; Ferrero, S.; Ceccarelli, C.; Ugliengo, P. Theoretical distribution of the ammonia binding energy at interstellar icy grains: a new computational framework. *ACS Earth and Space Chemistry* **2022**, *6*, 1514–1526.

(8) Cuppen, H. M.; Hornekær, L. Kinetic Monte Carlo studies of hydrogen abstraction from graphite. **2008**, *128*, 174707–174707.

(9) Cuppen, H. M.; Karssemeijer, L. J.; Lamberts, T. The Kinetic Monte Carlo Method as a

Way To Solve the Master Equation for Interstellar Grain Chemistry. *Chemical Reviews* **2013**, *113*, 8840–8871.
